# Extremozymes and compatible solute production potential of halophilic and halotolerant bacteria isolated from crop rhizospheric soils of Southwest Saurashtra Gujarat

**DOI:** 10.1038/s41598-024-63581-z

**Published:** 2024-07-08

**Authors:** Likhindra Reang, Shraddha Bhatt, Rukam Singh Tomar, Kavita Joshi, Shital Padhiyar, Hiren Bhalani, JasminKumar Kheni, U. M. Vyas, M. V. Parakhia

**Affiliations:** 1 Department of Biotechnology, Junagadh Agricultural University, Junagadh, Gujarat India; 2 Crop Improvement Section, ICAR - Directorate of Groundnut Research, Junagadh, Gujarat India; 3 Main Oilseed Research Station, Junagadh Agricultural University, Junagadh, Gujarat India

**Keywords:** Halophiles, Extremophile, 16S rRNA, Extremozyme, Ectoine, Glycine betaine, Biochemistry, Biotechnology, Microbiology

## Abstract

Halophiles are one of the classes of extremophilic microorganisms that can flourish in environments with very high salt concentrations. In this study, fifteen bacterial strains isolated from various crop rhizospheric soils of agricultural fields along the Southwest coastline of Saurashtra, Gujarat, and identified by 16S rRNA gene sequencing as *Halomonas pacifica*, *H. stenophila*, *H. salifodinae, H. binhaiensis, Oceanobacillus oncorhynchi,* and *Bacillus paralicheniformis* were investigated for their potentiality to produce extremozymes and compatible solute. The isolates showed the production of halophilic protease, cellulase, and chitinase enzymes ranging from 6.90 to 35.38, 0.004–0.042, and 0.097–0.550 U ml^−1^, respectively. The production of ectoine-compatible solute ranged from 0.01 to 3.17 mg l^−1^. Furthermore, the investigation of the ectoine-compatible solute production at the molecular level by PCR showed the presence of the ectoine synthase gene responsible for its biosynthesis in the isolates. Besides, it also showed the presence of glycine betaine biosynthetic gene betaine aldehyde dehydrogenase in the isolates. The compatible solute production by these isolates may be linked to their ability to produce extremozymes under saline conditions, which could protect them from salt-induced denaturation, potentially enhancing their stability and activity. This correlation warrants further investigation.

## Introduction

Extremophiles are organisms that can thrive in environments with extreme conditions such as extreme temperature, pressure, radiation, salinity, pH levels, etc.^1,2^ that have traditionally been considered inhospitable for life^3^. These organisms cope with harsh environments by adopting certain unique strategies for survival. For instance, the first reported extremophiles termed halophiles are known to have developed two classic strategies called ‘high-salt-in-cytoplasm’ and ‘salt-out-in-cytoplasm’ or ‘low-salt-high-compatible-solute-in-cytoplasm’ for osmoadaptation^4^. The former mechanism involves the accumulation of intracellular KCl concentrations higher than the external NaCl concentration to maintain the turgor pressure^5^. However, the resulting high ionic strength in their cytoplasm cost them a compensatory evolutionary change from normal to acidic proteome to keep the proteins soluble for maintaining normal functionality of key cellular activities thereby confining their adaptability to only hypersaline habitats without frequent fluctuations^6^ to typically 5 M NaCl or more, found mostly in extreme halophiles such as *Halobacteriales* (archaea), *Salinibacter ruber* (bacteria) and *Haloanaerobiales* (anaerobic moderate halophilic bacteria)^5,7^. Although energetically more expensive, the latter strategy employs a physiologically much more flexible mechanism involving either an accumulation of high concentrations of organic compatible solutes in the cytoplasm from external environments or their de-novo synthesis^7^ for osmotic adjustments thereby circumventing the long-lasting large-scale accumulation of ions^6^. This strategy facilitates the adaptability of the organisms possessing them to a wide range of salinities (typically 0.5–3 M NaCl). It is found largely in halotolerant and moderate halophiles^5^. These halophilic extremophiles also produce a special and unique enzyme called extremozymes to survive in intolerably hostile environments^3^. These extremozymes are known for their promising capability to withstand unusual extreme conditions required in industrial product synthesis processes where the mesophilic enzymes usually precipitate or denature^8^. They reportedly replace chemical catalysts in many industries, such as manufacturing chemicals, textiles, pharmaceuticals, detergents, food, paper, etc.^9,10^. Halophiles, reportedly one of the most important groups of such extremophiles are microorganisms that can flourish in environments with very high salt concentrations. They include members of all three domains of life, viz. Archaea, Bacteria, and Eukarya. In contrast, bacteria that can tolerate relatively high NaCl concentrations and grow regardless of salt's presence or absence are labeled halotolerant^11^.

Halophilic microorganisms have been reported as an excellent source of extremozyme called halozymes that can function stably under high salt concentrations and withstand high temperatures, alkaline pH, toxicants, etc. encountered in many industrial bioconversion processes^12^. The polyextremophilic nature of their enzymes makes halophiles a potential candidate for meeting the current industrial enzyme demands. Many scientists have reported their stability and dynamic performance in multiple extreme conditions, such as low water activity environments, aqueous/organic and non-aqueous solvents or media, etc.^8,13^. Such novel halozymes reported in halophiles include proteases, amylases, lipases, xylanases, nucleases, cellulases, catalases, and esterases^8^.

The ability of these halophiles to produce beneficial lysis enzymes such as chitinase, cellulase, and protease implies their direct potential use as biocontrol agents for controlling phytopathogenic fungi, pests, nematodes and for rhizospheric soil decomposition for increasing plant nutrient availability for sustainable agriculture and as livestock feed additives, etc.^14^. Besides, they also find applications in many industries such as food and feed, laundry and detergent, leather and textiles, pulp and paper, alcohol and beverages, medicine and pharmaceuticals, environmental bioremediations, biomass conversions for biofuel production, etc. So, given their profound agricultural and industrial significance, these enzymes viz., chitinase, cellulase, and protease were chosen in this study to unlock their production potential within the halophilic and halotolerant bacterial isolates to unveil and shed light on their vast potential applications.

These industrial enzymes are found in various sources, such as plants, animals, and microorganisms. However, microbial sources are preferred due to their high stability, cost-effectiveness, less time and space requirement, high consistency, production and optimization ease, and increasing demand in many industries^15^, as evidenced by their total contribution of more than 82% of revenue share in 2022^16^. However, despite these enzymes being widely studied in many organisms, only a few reports have been made on the extracellular enzymes of halophilic and halotolerant bacteria, especially *Halomonas* sp.^17^. Besides, halophiles have been isolated and investigated for many other possible biotechnological applications, such as the production of compatible solutes, enhanced oil recovery, and the degradation of industrial pollutants in saline habitats and as potential agricultural bioinoculants for the recovery of saline soils^11,18^.

Compatible solutes or osmoprotectants are low molecular weight organic molecules with a neutral charge and low toxicity at high concentrations either accumulated from external environments or secreted by halophiles in their cytoplasm to act as osmolytes for their survivability against the extreme osmotic stresses^19^. They include several different classes, such as high water-soluble sugars, alcohols or polyols, betaines, amino acids, ectoine, and its derivatives, among which ectoine and glycine-betaine are reported as the most predominant ones. They are used in many biotech industries for stabilizing enzymes, DNA, and whole cells against freezing and thawing, drying and heating, and denaturants such as urea and salts, and as salt antagonists, stress-protective agents, moisturizers, therapeutics, and for increasing the freshness of foods in food industries^20,21^. In plants, their accumulation is said to increase survivability against various stresses such as salinity, heat, and drought. The genetic manipulation of these osmoprotectants' responsive genes has been suggested as one of the strategies to improve plant stress tolerance by enhancing their production^22–24^.

In light of the above perspectives, the current research was conducted to study the production potentiality of extracellular halozymes viz. protease, cellulase, and chitinase, and ectoine compatible solute, and PCR based molecular detection of the biosynthetic gene of ectoine and glycine betaine of halophilic and halotolerant bacteria isolated from the crop rhizospheric soils of agricultural fields of southwest coastline of Saurashtra Gujarat.

## Results

### Preliminary soil analysis

The preliminary soil analysis results are summarized in Table 1. The physicochemical properties of the soil samples, including pH, electrical conductivity (E.C.), organic carbon content, and availability of phosphorous and potash ranged from 7.4 to 8.1, 0.76–1.59 dS m^−1^, 4.03–7.47 g kg^−1^, 29.57–54.33 kg ha^−1^ and 166.70–248.33 kg ha^−1^ respectively^11^.

**Table 1:** Physico-chemical characteristics of soil samples.

Sample no.	pH	EC (dS m^−1^)	OC (g Kg^−1^)	Available P_2_O_5_ (Kg ha^−1^)	Available K_2_O (Kg ha^−1^)
1	7.83	1.05	4.88 ± 0.01	29.57 ± 0.26	166.70 ± 1.65
2	7.92	0.81	7.46 ± 0.02	34.33 ± 0.11	201.24 ± 0.10
3	7.90	1.32	5.50 ± 0.02	37.75 ± 0.11	211.36 ± 0.13
4	7.47	1.24	4.73 ± 0.01	36.07 ± 0.69	207.48 ± 1.00
5	8.10	0.79	6.40 ± 0.03	39.46 ± 0.16	209.35 ± 0.10
6	8.13	1.00	4.03 ± 0.02	45.50 ± 0.08	221.71 ± 0.10
7	8.15	1.13	6.73 ± 0.02	49.47 ± 0.14	233.36 ± 0.16
8	8.02	1.09	6.20 ± 0.02	45.81 ± 1.27	223.39 ± 2.25
9	8.04	1.10	6.80 ± 0.02	49.14 ± 0.12	232.51 ± 0.17
10	7.96	1.57	5.50 ± 0.02	48.53 ± 0.06	231.30 ± 0.17
11	7.91	0.76	6.93 ± 0.01	49.01 ± 0.14	235.64 ± 0.13
12	7.89	1.59	4.50 ± 0.01	51.51 ± 0.14	247.25 ± 0.10
13	8.06	1.16	5.03 ± 0.01	54.33 ± 0.12	248.32 ± 0.13
14	7.97	1.13	4.73 ± 0.01	51.76 ± 1.37	233.88 ± 5.78
15	8.10	0.77	4.66 ± 0.01	53.64 ± 0.10	247.74 ± 0.13
S.Em. ±			0.01	0.52	2.05
C.D. at 5%			0.05	1.52	5.94
C.V. %			5.60	2.03	1.59

OC, organic carbon; values of OC, P_2_O_5,_ and K_2_O are mean ± standard error of three replicates.

### Halophilic characterization

The characterization of the isolates by NaCl tolerance test showed that the isolates could tolerate up to 25% with optimum growth between 10 and 15% NaCl concentrations^11^. The isolates S_1_ through S_9_ and S_11_, all belonging to the *Halomonas* species, exhibited remarkable and robust growth despite challenging salt concentrations exceeding 10–15% NaCl. These *Halomonas* isolates surpassed their counterparts S_10_, S_12_, S_13_, S_14_, and S_15_ belonging to the *Oceanobacillus* and *Bacillus* species with exceptional vigor. Intriguingly, the *Halomonas* species isolates maintained a consistently thriving growth pattern even at higher NaCl concentrations, with only a modest decline observed up to 25% NaCl. This remarkable resilience and adaptability demonstrated by the *Halomonas* isolates highlight their exceptional ability to thrive in extremely saline environments. In stark contrast, the *Oceanobacillus* and *Bacillus* species isolates, S_10_, S_12_, S_13_, S_14_, and S_15_, exhibited a stark decline in their growth trajectory, indicating their limited capacity for salt tolerance and a considerably less robust response to the challenging conditions presented by 25% NaCl concentration.

### Microscopic characterization

**Figure 1:**
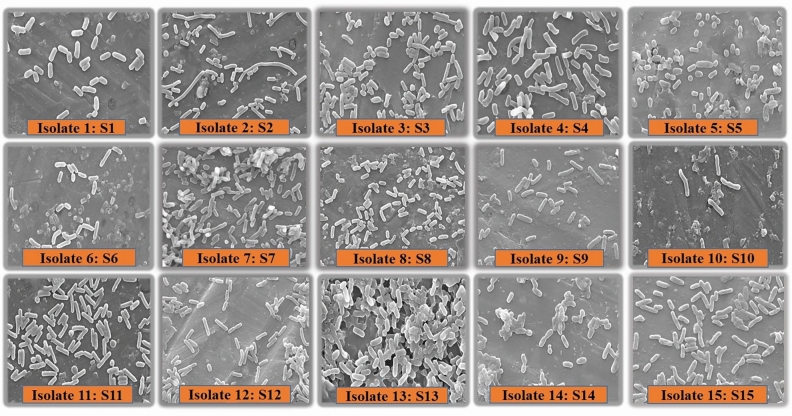
Microscopic characterization of isolates by Scanning Electron Microscope (SEM).

The gram staining analysis of the isolates revealed that all the *Halomonas* species isolates exhibited gram-negative characteristics, while the *Bacillus* species isolates exhibited gram-positive characteristics at the microscopic level. This result was complemented by the identification of isolates based on 16S rRNA partial gene sequencing. The scanning electron microscopic characterization identified the isolates as short to thin rod-shaped bacteria in single or pairs to bunchy type organization (Fig. 1) with sizes ranging from 0.38 to 0.83 μm by 0.75–6.78 μm^11^. All the isolates were motile, as observed under the microscope.

### Molecular identification and phylogenetic analysis

**Figure 2:**
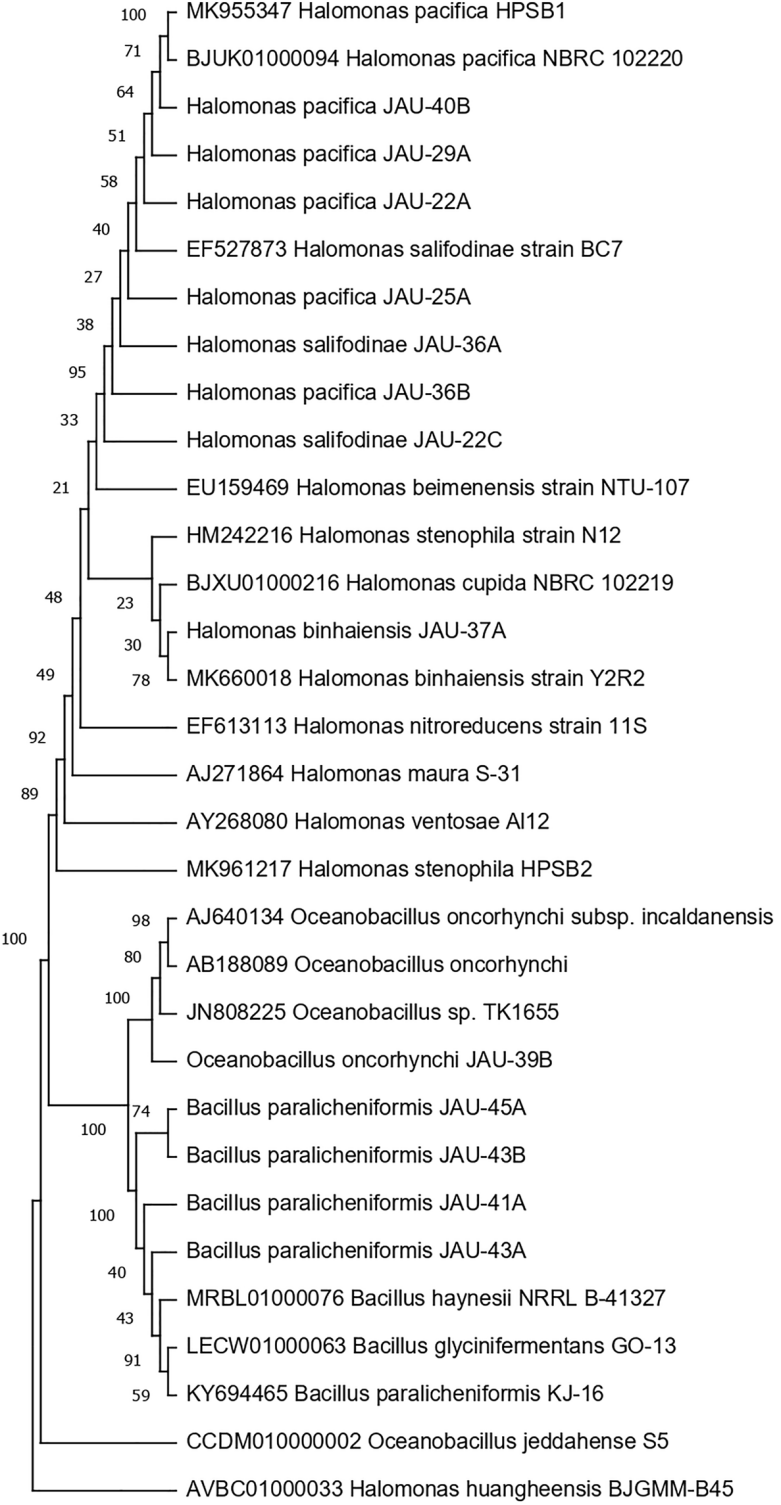
Phylogenetic dendogram, based on 16S rRNA nucleotide sequences, showing the genetic interrelationship among the different halophlic and halotolerant bacterial isolates within the closely related species of various genera.

The analysis of the partial 16S rRNA gene sequence of the isolates to determine their genetic relatedness and taxonomic identity by comparing their sequences to the known reference strains in the curated 16S database of EzBioCloud revealed that isolates S_1_, S_3_, S_5_, S_6_, S_8_, and S_11_ belonged to *Halomonas pacifica* with percent similarity of 99.15%, 99.45%, 99.52%, 99.45%, 99.01% and 99.52% with *H. pacifica* NBRC 102220 respectively (Table 2). In contrast, isolate S2 belonged to *Halomonas stenophila* with a percent similarity of 99.12% with *H. stenophila* N12. Isolates S_4_ and S_7_ belonged to *H. salifodinae* with percent similarity of 99.34% and 99.22% respectively while isolate S9 belonged to *Halomonas binhaiensis* with 99.52% similarity with *Halomonas binhaiensis* Y2R2 (Table 2). On the other hand, isolate S10 belonged to *Oceanobacillus oncorhynchi* with a percent similarity of 98.23% with *Oceanobacillus oncorhynchi* subsp. *oncorhynchi* R-2 while isolates S12, S13, S14 and S15 belonged to *Bacillus paralicheniformis* with percent similarity of 99.59%, 99.46%, 98.19% and 98.44% with *B. paralicheniformis* KJ-16 (Table 2). The partial 16S rRNA gene sequence of isolates S1 and S2 were submitted to NCBI with accession numbers MK955347 and MK961217 re-designating the isolates as *Halomonas pacifica* HPSB1 and *Halomonas stenophila* HPSB2 respectively^11^. The BLAST analysis demonstrated the highest level of genetic relations between the respective *Halomonas*, *Oceanobacillus*, and *Bacillus* isolates which features and supports their taxonomic uniformity within the genus. The phylogenetic analysis of these isolates, along with their genetically closest reference species, constructed using MEGA11 following the Minimum Evolution tree method using their multiple sequence alignment (MSA) aligned by ClustalW, and analyzed based on the maximum composite likelihood substitution model, distinctly elucidated the interconnectedness among various genera within their respective species, as visually depicted in Fig. 2.

**Table 2:** Determination of percent similarity and identification of isolates by 16S rRNA gene sequence alignment in EzBioCloud BLAST.

Isolate no.	Strain designated	Significant alignment with most closely related organisms^a^			
		Accession ID	Hit taxon and strain name	Query coverage %	Identity %
1	*Halomonas pacifica* strain_JAU-7B	BJUK01000094	*Halomonas pacifica* NBRC 102220(T)	100%	99.15%
		EF527873	*Halomonas salifodinae* BC7(T)	97.7%	98.67%
		EU159469	*Halomonas beimenensis* NTU-107(T)	96.9%	96.72%
		AY268080	*Halomonas ventosae* Al12(T)	95.8%	96.60%
		MK660018	*Halomonas binhaiensis* Y2R2(T)	100%	96.49%
2	*Halomonas stenophila* strain_JAU-20A	HM242216	*Halomonas stenophila* N12(T)	100%	99.12%
		EF527873	*Halomonas salifodinae* BC7(T)	97.7%	98.80%
		AJ271864	*Halomonas maura* S-31(T)	95.6%	98.73%
		BJUK01000094	*Halomonas pacifica* NBRC 102220(T)	100%	98.67%
		EF613113	*Halomonas nitroreducens* 11S(T)	98.7%	98.49%
3	*Halomonas pacifica* strain_ JAU-22A	BJUK01000094	*Halomonas pacifica* NBRC 102220(T)	100%	99.45%
		EF527873	*Halomonas salifodinae* BC7(T)	97.7%	99.30%
		HM242216	*Halomonas stenophila* N12(T)	100%	97.88%
		MK660018	*Halomonas binhaiensis* Y2R2(T)	100%	97.74%
		AY268080	*Halomonas ventosae* Al12(T)	95.8%	97.63%
4	*Halomonas pacifica* strain_JAU-22C	EF527873	*Halomonas salifodinae* BC7(T)	97.7%	99.34%
		BJUK01000094	*Halomonas pacifica* NBRC 102220(T)	100%	99.27%
		AY268080	*Halomonas ventosae* Al12(T)	95.8%	97.68%
		HM242216	*Halomonas stenophila* N12(T)	100%	97.61%
		MK660018	*Halomonas binhaiensis* Y2R2(T)	100%	97.47%
5	*Halomonas pacifica* strain_JAU-25A	BJUK01000094	*Halomonas pacifica* NBRC 102220(T)	100%	99.52%
		EF527873	*Halomonas salifodinae* BC7(T)	97.7%	99.37%
		HM242216	*Halomonas stenophila* N12(T)	100%	97.95%
		MK660018	*Halomonas binhaiensis* Y2R2(T)	100%	97.81%
		AY268080	*Halomonas ventosae* Al12(T)	95.8%	97.71%
6	*Halomonas pacifica* strain_JAU-29A	BJUK01000094	*Halomonas pacifica* NBRC 102220(T)	100%	99.45%
		EF527873	*Halomonas salifodinae* BC7(T)	97.7%	99.30%
		HM242216	*Halomonas stenophila* N12(T)	100%	97.88%
		MK660018	*Halomonas binhaiensis* Y2R2(T)	100%	97.74%
		AY268080	*Halomonas ventosae* Al12(T)	95.8%	97.63%
7	*Halomonas pacifica* strain_JAU-36A	EF527873	*Halomonas salifodinae* BC7(T)	97.7%	99.22%
		BJUK01000094	*Halomonas pacifica* NBRC 102220(T)	100%	99.09%
		AY268080	*Halomonas ventosae* Al12(T)	95.8%	97.53%
		HM242216	*Halomonas stenophila* N12(T)	100%	97.47%
		MK660018	*Halomonas binhaiensis* Y2R2(T)	100%	97.34%
8	*Halomonas pacifica* strain_JAU-36B	BJUK01000094	*Halomonas pacifica* NBRC 102220(T)	100%	99.01%
		EF527873	*Halomonas salifodinae* BC7(T)	97.7%	98.93%
		HM242216	*Halomonas stenophila* N12(T)	100%	97.38%
		MK660018	*Halomonas binhaiensis* Y2R2(T)	100%	97.24%
		AY268080	*Halomonas ventosae* Al12(T)	95.8%	97.22%
9	*Halomonas stenophila* strain_JAU-37A	MK660018	*Halomonas binhaiensis* Y2R2(T)	100%	99.52%
		AVBC01000033	*Halomonas huangheensis* BJGMM-B45(T)	100%	98.84%
		BJXU01000216	*Halomonas cupida* NBRC 102219(T)	100%	98.15%
		HM242216	*Halomonas stenophila* N12(T)	100%	98.02%
		AY268080	*Halomonas ventosae* Al12(T)	95.8%	97.78%
10	*Oceanobacillus aidingensis* strain_JAU-39B	AB188089	*Oceanobacillus oncorhynchi subsp. oncorhynchi* R-2(T)	95.4%	98.23%
		AJ640134	*Oceanobacillus oncorhynchi* subsp. *incaldanensis* 20AG(T)	100%	98.09%
		JN808225	*Oceanobacillus gochujangensis* TK1655(T)	100%	97.36%
		CCDM010000002	*Oceanobacillus jeddahense* S5(T)	100%	97.36%
		LT727813	LT727813_s SK-2	100%	97.06%
11	*Halomonas pacifica* strain_JAU-40B	BJUK01000094	*Halomonas pacifica* NBRC 102220(T)	100%	99.52%
		EF527873	*Halomonas salifodinae* BC7(T)	97.7%	99.37%
		HM242216	*Halomonas stenophila* N12(T)	100%	97.95%
		MK660018	*Halomonas binhaiensis* Y2R2(T)	100%	97.81%
		AY268080	*Halomonas ventosae* Al12(T)	95.8%	97.71%
12	*Bacillus haynesii* strain_JAU-41A	KY694465	*Bacillus paralicheniformis* KJ-16(T)	100%	99.59%
		LECW01000063	*Bacillus glycinifermentans* GO-13(T)	100%	99.39%
		MRBL01000076	*Bacillus haynesii* NRRL B-41327(T)	100%	99.32%
		AYTN01000016	*Bacillus sonorensis* NBRC 101234(T)	100%	99.18%
		AE017333	*Bacillus licheniformis* ATCC 14580(T)	100%	99.12%
13	*Bacillus licheniformis* strain_JAU-43A	KY694465	*Bacillus paralicheniformis* KJ-16(T)	100%	99.46%
		LECW01000063	*Bacillus glycinifermentans* GO-13(T)	100%	99.25%
		MRBL01000076	*Bacillus haynesii* NRRL B-41327(T)	100%	99.18%
		AE017333	*Bacillus licheniformis* ATCC 14580(T)	100%	99.12%
		AYTN01000016	*Bacillus sonorensis* NBRC 101234(T)	100%	99.05%
14	*Bacillus haynesii* strain_JAU-43B	KY694465	*Bacillus paralicheniformis* KJ-16(T)	100%	98.19%
		MRBL01000076	*Bacillus haynesii* NRRL B-41327(T)	100%	98.05%
		LECW01000063	*Bacillus glycinifermentans* GO-13(T)	100%	97.98%
		AE017333	*Bacillus licheniformis* ATCC 14580(T)	100%	97.84%
		AUQZ01000032	AUQZ_s NSP9.1	100%	97.77%
15	*Bacillus haynesii* strain_JAU-45A	KY694465	*Bacillus paralicheniformis* KJ-16(T)	100%	98.44%
		LECW01000063	*Bacillus glycinifermentans* GO-13(T)	100%	98.23%
		MRBL01000076	*Bacillus haynesii* NRRL B-41327(T)	100%	98.17%
		AYTN01000016	*Bacillus sonorensis* NBRC 101234(T)	100%	98.03%
		AE017333	*Bacillus licheniformis* ATCC 14580(T)	100%	97.96%

^a^Data obtained after BLAST analysis from EzBioCloud 16S database.

### Quantification of protease, cellulase, and chitinase enzymes

The results of the quantitative protease, cellulase, and chitinase enzyme assays and the respective enzymes’ specific activities of the isolates are presented in Supplementary Tables 1, 2, and 3, respectively. The various standard curves viz*.* tyrosine, glucose, NAG, and BSA used to estimate the protease, cellulase, and chitinase enzymes, and protein content of the isolates in the respective enzyme production medium are shown in Supplementary Figs. 1, 2, 3, and 4 respectively.

**Figure 3:**
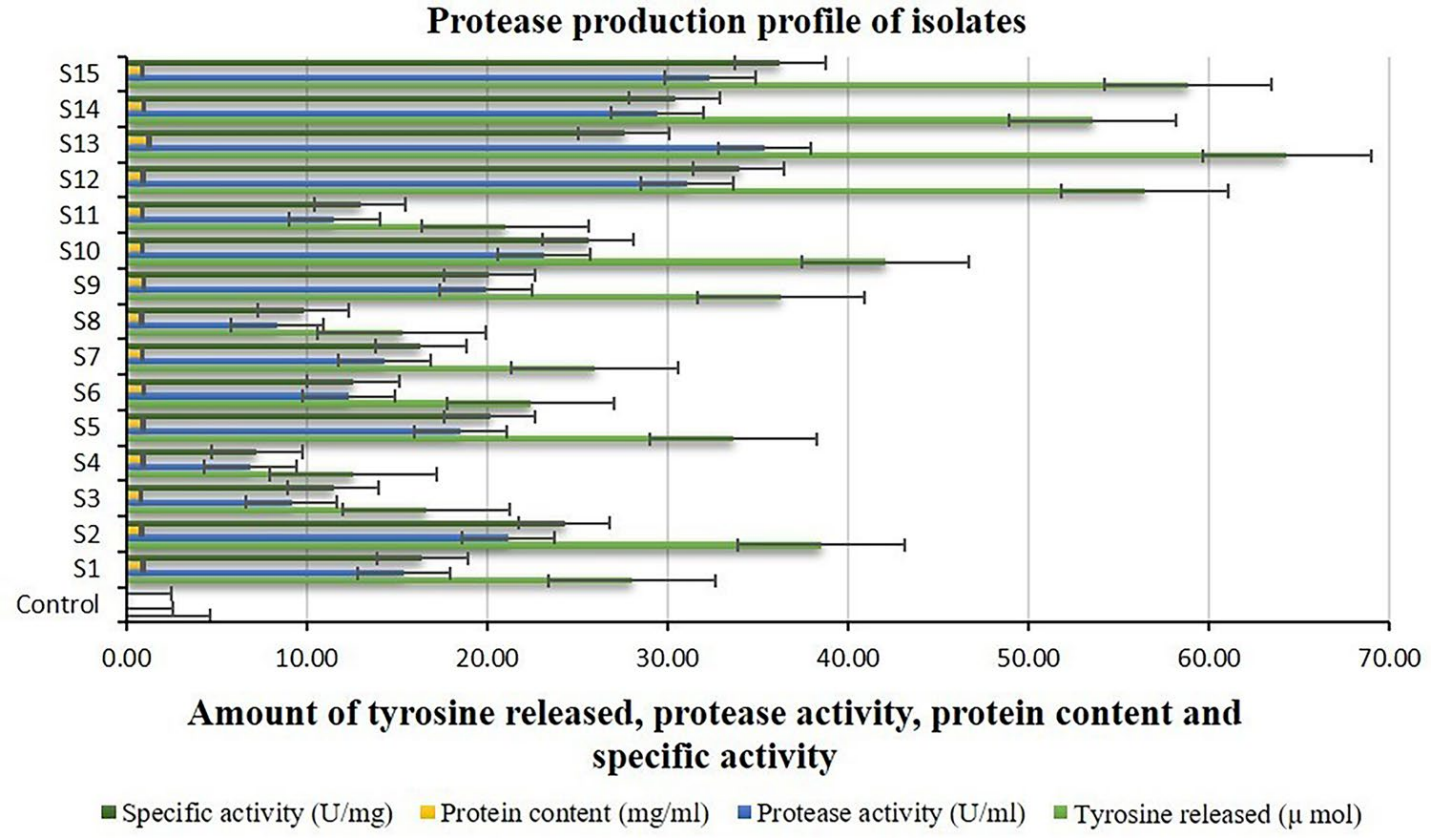
Protease activity of halophilic and halotolerant bacterial isolates (U ml^−1^ min^−1^).

**Figure 4:**
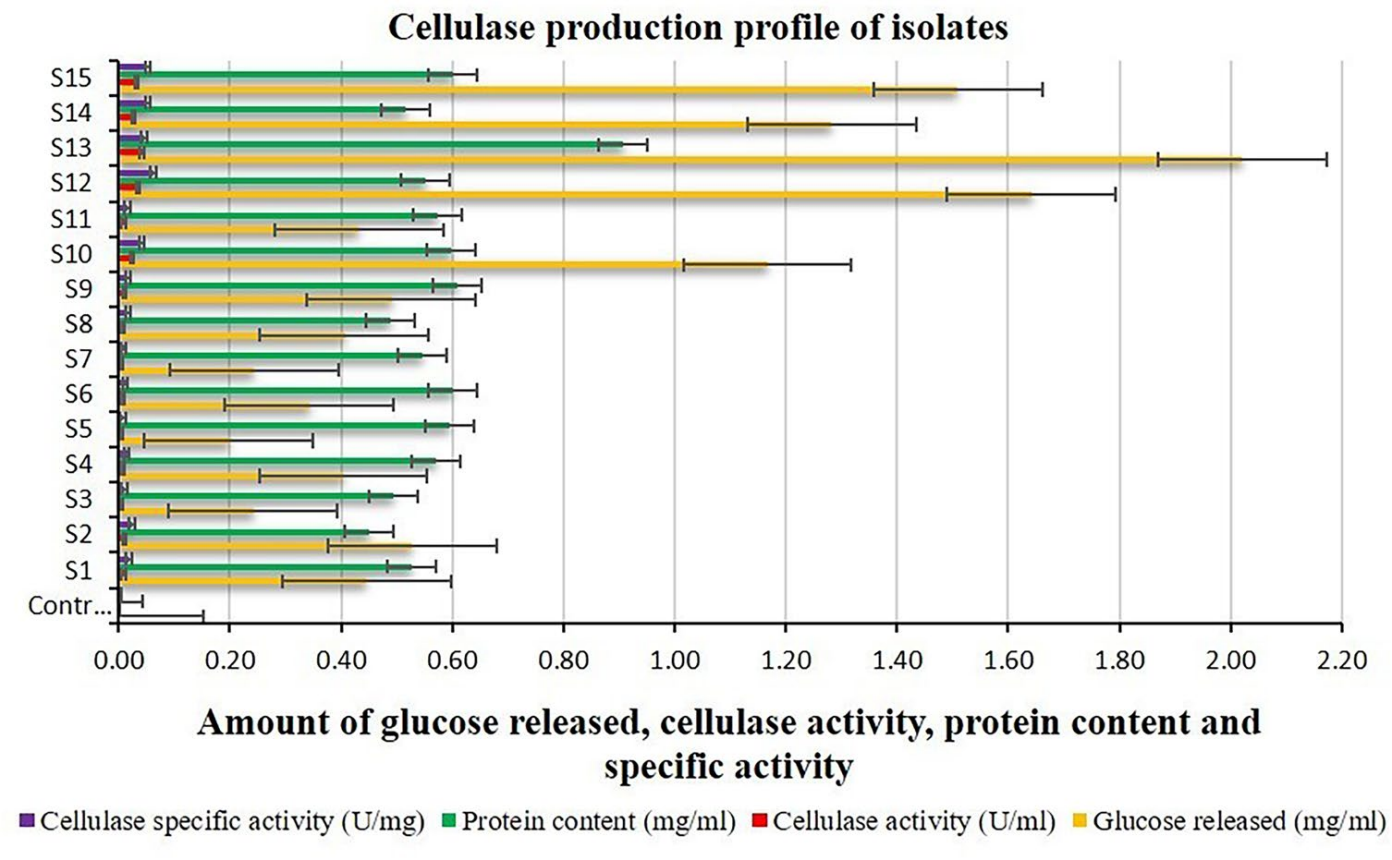
Cellulase activity of halophilic and halotolerant bacterial isolates (U ml^−1^ min^−1^).

**Figure 5:**
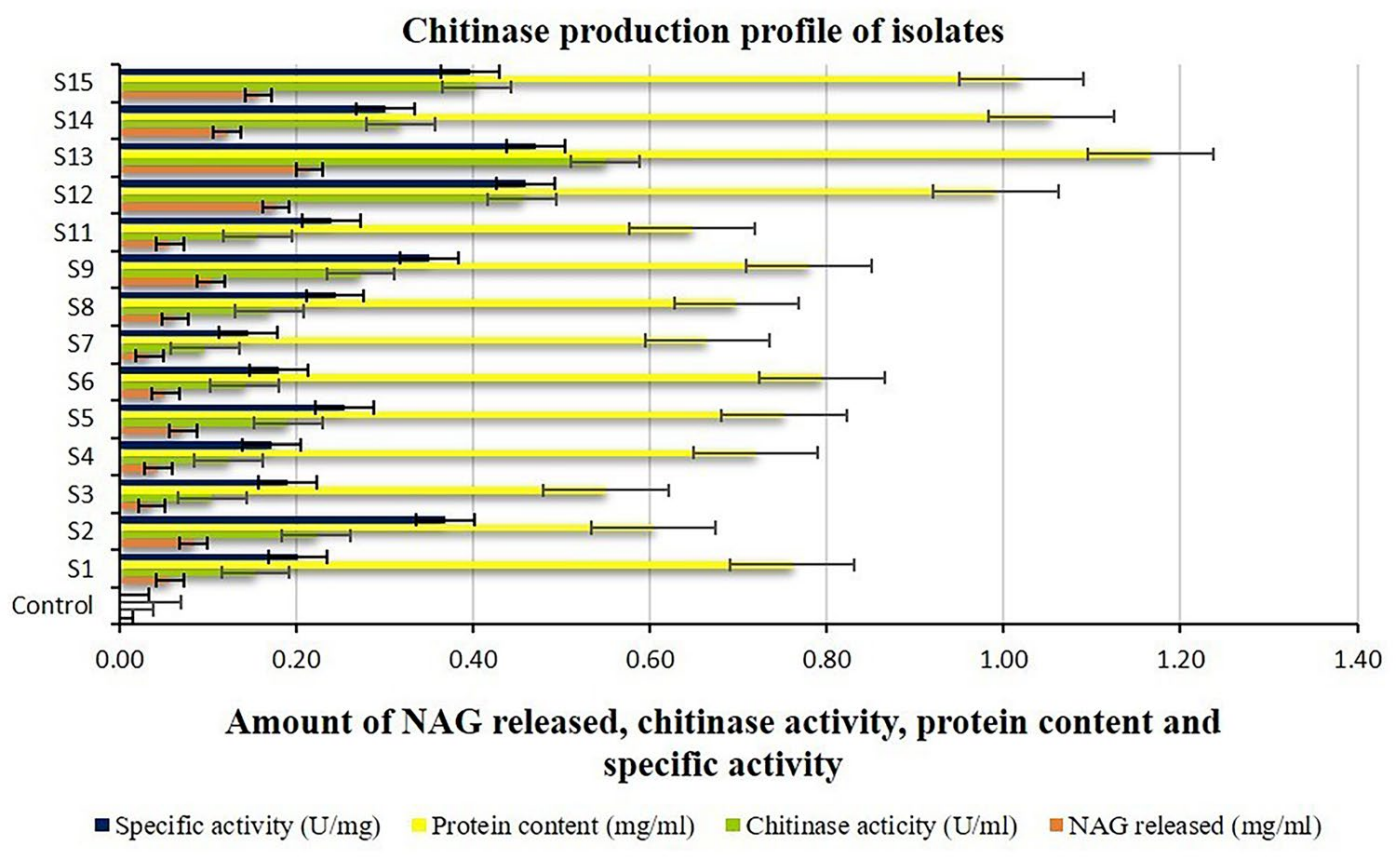
Chitinase and its specific activity and protein content of halophilic and halotolerant bacterial isolates.

The protease, cellulase, and chitinase activities of the isolates ranged from 6.90 to 35.38 U ml^−1^ min^−1^, 0.004–0.042 U ml^−1^ min^−1^, and 0.097–0.550 U ml^−1^ h^−1^ respectively, while their respective corresponding specific activities ranged from 7.23 to 36.21 U mg^−1^ min^−1^, 0.007–0.062 U mg^−1^ min^−1^, and 0.146–0.471 U mg^−1^ h^−1^, respectively. The highest protease, cellulase, and chitinase activities were shown by isolate S13, while the lowest was shown by isolates S4 (Fig. 3), S5 (Fig. 4), and S7 (Fig. 5), respectively. On the other hand, the highest protease, cellulase, and chitinase-specific activities were shown by isolates S15 (Fig. 3), S12 (Fig. 4), and S13 (Fig. 5), respectively while the lowest was shown by isolates S4, S5, and S7, respectively.

The protein content of the isolates in their respective protease, cellulase, and chitinase enzyme production medium ranged from 0.80 to 1.28 mg ml^−1^, 0.450–0.908 mg ml^−1^, and 0.550 to 1.166 mg ml^−1^, respectively. Isolate S13 exhibited the highest protein content in the protease, cellulase, and chitinase enzyme production medium, while isolates S3 (Fig. 3), S2 (Fig. 4), and S3 (Fig. 5), respectively, showed the lowest protein content. The statistical analysis of variance (ANOVA) for all the data of tyrosine, glucose, and NAG released, protease, cellulase, and chitinase activity, protein contents, and respective enzymes’ corresponding specific activities of the isolates indicated a high level of significance inferred from the greater value of calculated F than that of table F at both the 1% and 5% significance levels.

### Ectoine production potentiality

**Figure 6:**
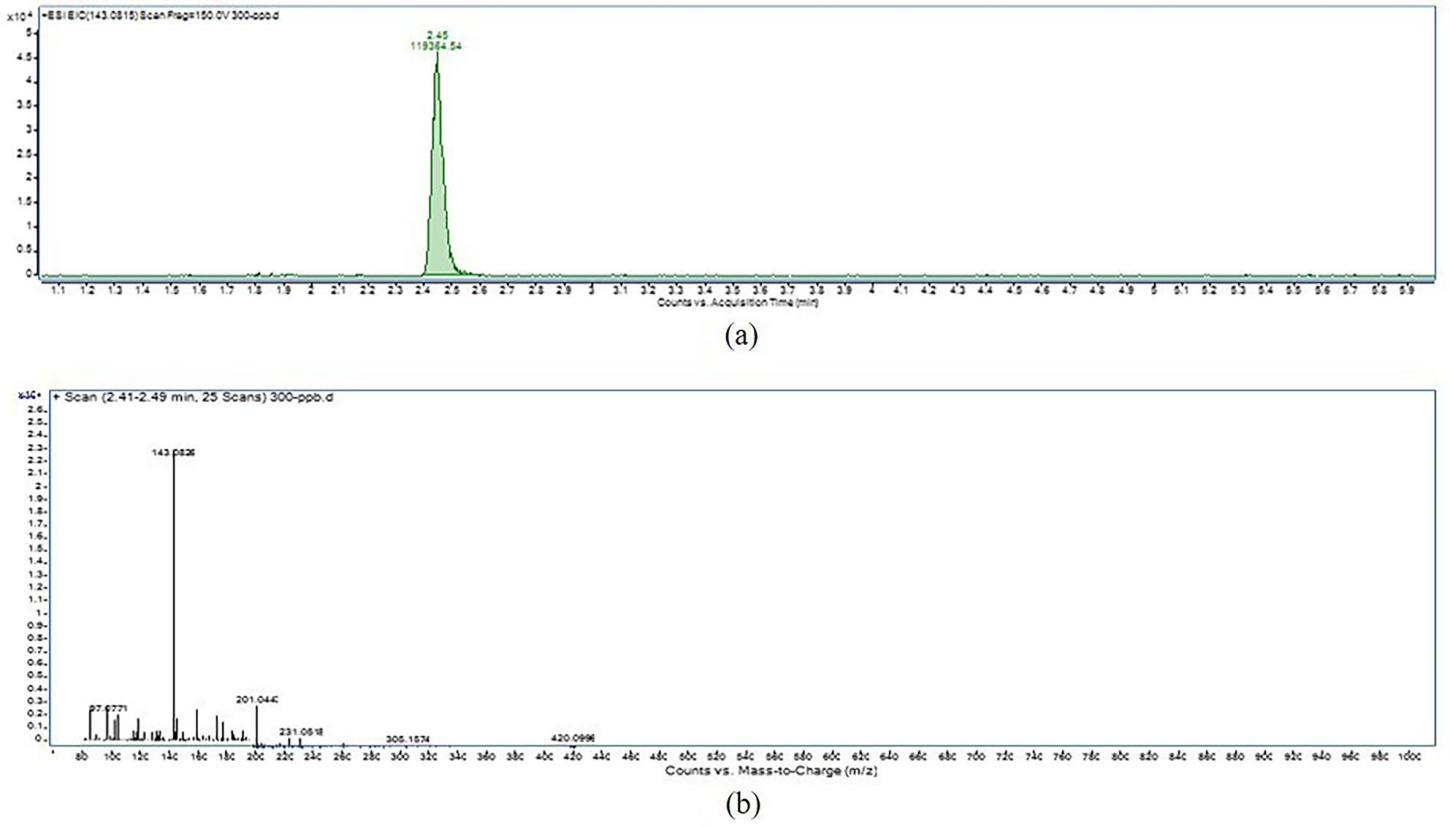
(**a**) Chromatogram, and (**b**) mass spectrum profile of ectoine standard.

**Figure 7:**
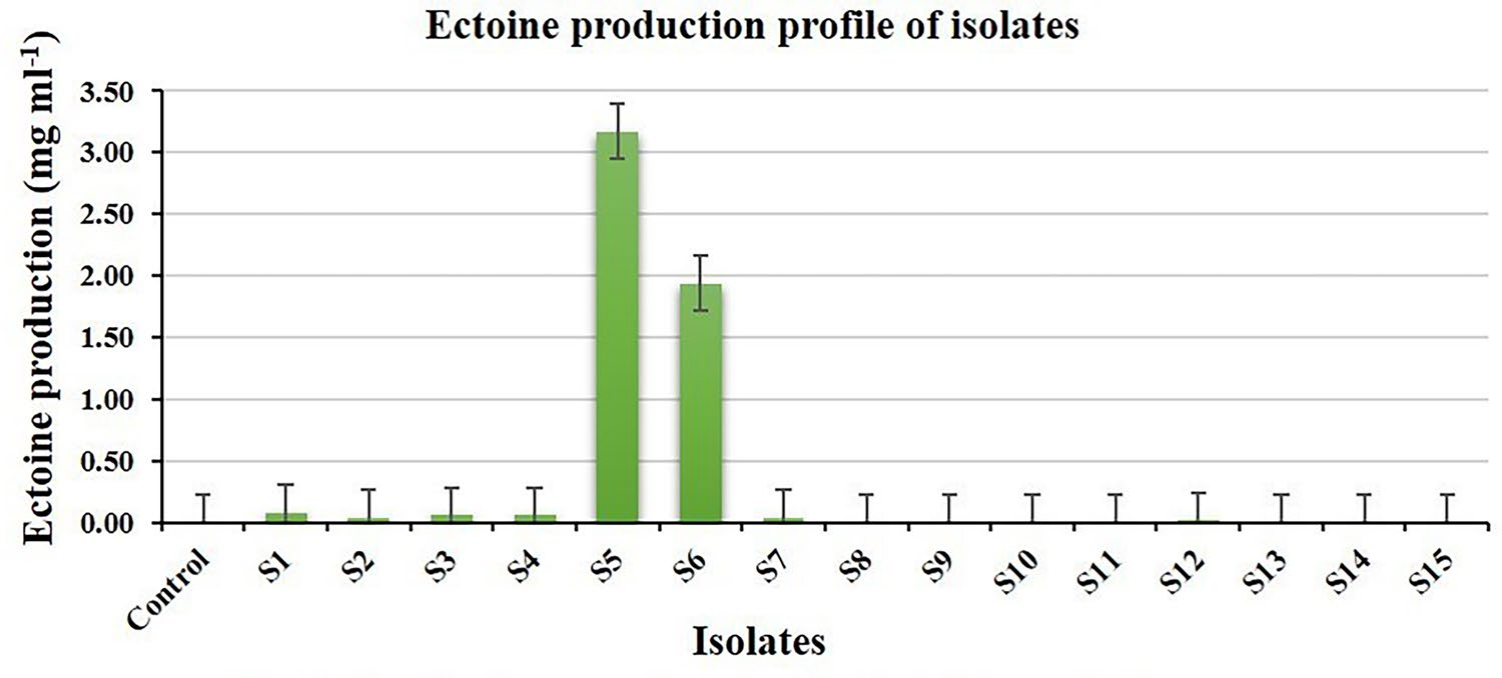
Production profile of ectoine by different isolates.

**Figure 8:**
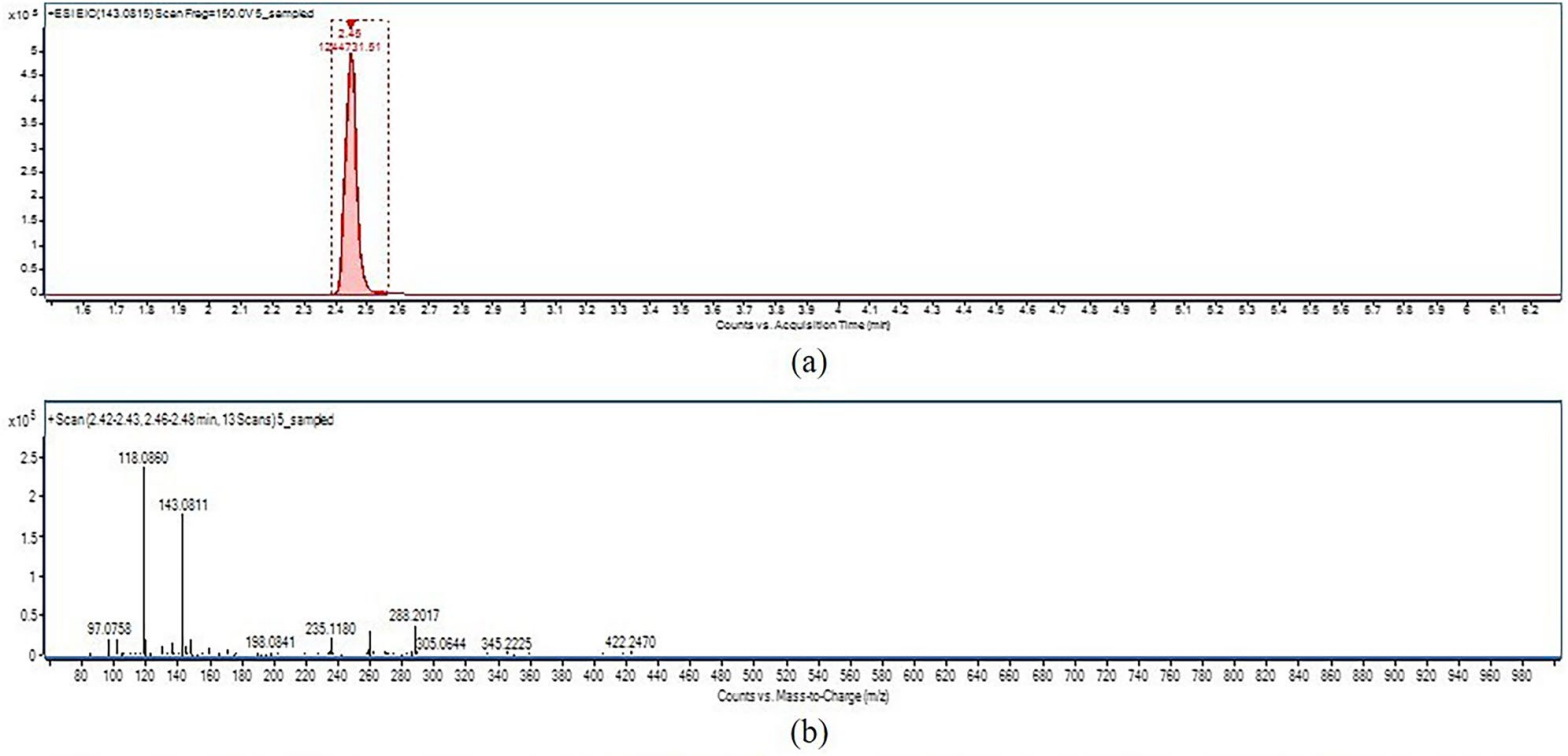
(**a**) Chromatogram, and (**b**) mass spectrum profile of highest ecoine production by isolate S_5_.

The results of the ectoine production potentiality of isolates are presented in Table 3. The ectoine production of the isolates was determined according to the peak generated by the ectoine standard at varying concentrations in LCMS (Fig. 6). The ectoine production ranged from 0.01 to 3.17 mgl^−1^ shown by the isolates S9 and S10 and S5, respectively (Fig. 7). The chromatogram and mass spectrum profile of the highest ectoine production by the isolate S5 is shown in Fig. 8. However, five out of fifteen isolates showed no detectable ectoine production, perhaps due to deficient ectoine production below the detection threshold limit as confirmed by the presence of their ectoine biosynthetic gene described below in PCR based molecular detection of ectoine biosynthetic gene.

**Table 3:** Ectoine production of isolates.

Sr. no.	Isolate code	RT	Ectoine production	
			ppb	Mg l^−1^
1	S1	2.45	84.41	0.08 ± 0.017
2	S2	2.43	35.83	0.04 ± 0.012
3	S3	2.46	55.54	0.06 ± 0.006
4	S4	2.44	55.86	0.06 ± 0.006
5	S5	2.45	3168.06	3.17 ± 0.010
6	S6	2.46	1943.56	1.94 ± 0.012
7	S7	2.47	36.76	0.04 ± 0.006
8	S8	ND	ND	ND
9	S9	2.47	8.42	0.01 ± 0.001
10	S10	2.47	9.55	0.01 ± 0.002
11	S11	ND	ND	ND
12	S12	2.47	24.39	0.02 ± 0.006
13	S13	ND	ND	ND
14	S14	ND	ND	ND
15	S15	ND	ND	ND
16	Control	ND	ND	ND
S.Em. ±			0.007	
C.D. at 5%			0.020	
C.V. %			3.618	

Keys: Values of ectoine production are mean ± standard error of three replicates.

RT, retention time; ND, not detected.

### PCR amplification of ectoine and glycine betaine biosynthetic genes

**Figure 9:**
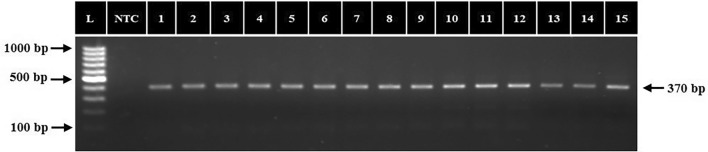
PCR amplification of *ectC* gene in halophilic and halotolerant bacterial isolates. L: 100 bp DNA ladder; NTC: no template control; 1–15: bacterial isolates.

**Figure 10:**
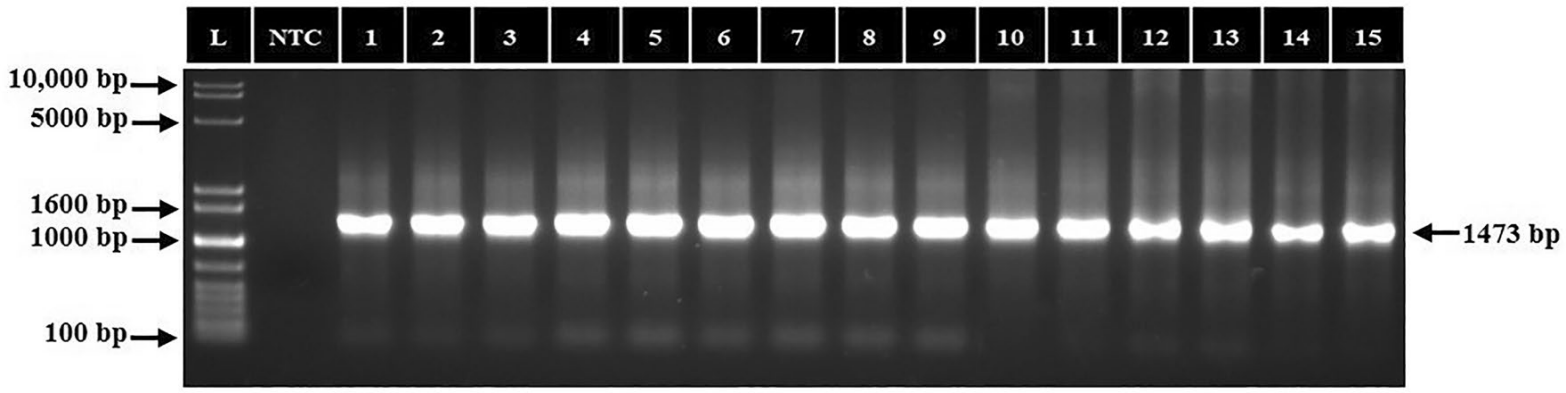
PCR amplification of *BADH1* gene in halophilic and halotolerant bacterial isolates. L: 1 kb DNA ladder; NTC: no template control; 1–15: bacterial isolates.

The PCR amplification targeting the *ectC *and *BADH1* genes confirmed their presence in all fifteen isolates, yielding amplicons of 370 bp (Fig. 9) and 1473 bp (Fig. 10), closely aligning with the sizes reported by Rajan et al*.*^25^ for *ectC* and Anburajan et al*.*^26^ for *BADH1*. The PCR-based molecular detection of the *ectC* gene thus confirmed and validated the positive ectoine production result obtained by QTOF LCMS as described above.

## Discussion

The NaCl tolerance test revealed that the isolates were moderate halophiles and halotolerant in nature based on the concentration of salt required for optimum growth and their maximum salt tolerance capacity as per the most widely accepted classification by Kushner and Kamekura^11,27^. This finding was further confirmed and validated by the molecular identification of the isolates by 16S rRNA gene sequencing at genus and species levels. The 16S rRNA gene sequence of the isolates belonging to *H. pacifica* and *H. stenophila* were submitted to NCBI with accession numbers MK955347 and MK961217, respectively^11^.

The protease activity of the isolates belonging to *Halomonas* species was approximately twofold higher than that of *H. meridian* HC4321C1 reported by Anithajothi et al.^28^. For those isolates belonging to *Bacillus* and *Oceanobacillus* species, the protease activity was in line with that of *B. licheniformis* P003 and *Oceanobacillus aidingensis*, reported by Sarker et al.^29^ and Kumar et al*.*^13^, respectively. However, the findings of many reports suggest that *B. licheniformis* strains are capable of producing much higher protease activity ranging above 100 to more than a few 1000 U ml^−1^^30–32^ depending upon the degree of culture enrichment with different sources of nitrogen, carbon, and substrates, etc., and is supposedly reported as one of the industrial strain of choice for enzyme production. The protease activity of *H. pacifica* and *H. stenophila *is being reported for the first time in our study. The optimum enzyme production potentiality of any microorganism is said to depend on the degree of optimized conditions for many factors such as pH, temperature, incubation period, agitation rate, substrate type, sources of carbon, nitrogen, etc., therefore, subject to vary from genus to genus and species to species. These results thus suggest that the isolates under study present suitable candidates for producing protease enzymes for various biotechnological and industrial applications.

The cellulase activity of the isolates belonging to *Halomonas* species was found to agree with the report of Shivanand et al.^12^ but much less than that of *Halomonas* sp. PV1 reported by Benit et al.^33^. In contrast, the isolate belonging to *Bacillus paralicheniformis* showed slightly less than the lowest cellulase activity of the same species reported by da Silva et al.^34^. However, the cellulase activity of the isolates belonging to *Bacillus* species was at least tenfold higher than that of isolates belonging to *Halomonas* species. These findings imply that *Bacillus* species have higher cellulase production potential than those *Halomonas* species. On the other hand, the cellulase activity of the isolate belonging to *Oceanobacillus oncorhynchi* was nearly tenfold lesser than that of *Oceanobacillus profundus* reported by Gbenro et al.^35^. Nevertheless, the ability of the isolates to produce cellulase enzyme suggests their ability to degrade cellulose, thereby implying the need for further investigation to uncover their fullest potential for the production of the same on a larger scale by providing the best optimum production conditions and suitable infrastructure facilities.

The highest chitinase activity shown by the isolate belonging to *B. paralicheniformis* was found to agree with the report of Akhir et al.^36^. While it was approximately tenfold higher than that of *B. licheniformis* JP2 reported by Keliat et al*.*^37^, Hussin and Majid^38^ have reported even a much lesser chitinase activity of similar species. However, the highest chitinase activity observed in our isolates is still much lesser than that of similar species reported by scientists such as Akeed et al.^39^ and Sasi et al.^40^. On the other hand, the chitinase activity of halophilic bacterial species is very limited to date, specifically of *Halomonas* species, and is being reported for the first time in our study. Furthermore, few earlier reports on the chitinase activity of halophilic bacteria such as *Virgibacillus marismortui* M3-23^41^ and *Planococcus rifitoensis* M2-26^42^ do prove the chitinase production potentiality of halophilic bacteria. However, there are multiple reports on the chitinase activity of many halotolerant bacteria, particularly that of *Bacillus* species which is supposedly used as one of the commercial, industrial strains. Nonetheless, the ability of our isolates to produce chitinase enzyme does suggest their ability to degrade chitin compounds, thereby representing a potential biocontrol agent for sustainable agriculture besides various other applications in many industries.

The ectoine production by all the isolates was found to be negligible to significantly less as compared to that of different *Halomonas* species reported by Zhang et al*.*^43^, Van-Thuoc et al*.*^44^, Van-Thuoc et al*.*^45^, Chen et al*.*^46^, Chen et al*.*^47^ where ectoine production ranged from 3.65 to 13.96 g l^−1^. The ectoine production of the isolates belonging to *H. pacifica, H. stenophila, H. salifodinae*, *H. binhaiensis*, *O. oncorhynchi,* and *B. paralicheniformis* has not been reported earlier. It is being reported for the first time in our study.

The lower amount of ectoine production by the isolates may be attributed to the unoptimized rate of agitation, which is reported to impact oxygen transfer in which a higher agitation rate for some halophiles results in higher dissolved oxygen (DO) level and thereby higher ectoine production. In contrast, a higher agitation rate has also been reported to result in a high shearing force of agitation, lowering microbial growth and ectoine production at an agitation rate higher than 200 rpm^47^. Furthermore, Chen et al*.*^47^ achieved higher ectoine production potentiality after utilizing a well-optimized production system with the best carbon and nitrogen sources, optimum ratio, optimum NaCl concentration, and agitation rate. Nevertheless, the production of ectoine by the isolates, even though in smaller quantity, still implies their potentiality for the production of ectoine-compatible solute.

The *ectC* gene amplified in the isolates is reported to encode putative proteins of 129 amino acids and codes for the L-ectoine synthase protein that catalyzes the final step of the ectoine biosynthetic pathway leading to the synthesis of ectoine osmolyte^25^. On the other hand, the Betaine Aldehyde Dehydrogenase gene is reportedly encoded by a polynucleotide of 1473 bp (Fig. 10) and polypeptides of 490 amino acids^26^. The *BADH1* gene catalyzes the conversion of betaine aldehyde to glycine betaine in the last step of the biosynthetic pathway that leads to the synthesis of the effective compatible solute glycine betaine, which maintains the fluidity of membranes and protects the biological structure of the organisms under salt stress conditions^26^. Additionally, similar to ectoine, glycine betaine also aids in stabilizing key proteins like proteases, cellulases, and chitinases against salt-induced denaturation, which are essential for nutrient acquisition and energy metabolism from organic substrates in extreme environments. The presence of the *BADH1* gene in the halophilic and halotolerant bacterial isolates suggests their potential to produce glycine betaine, expanding their repertoire of compatible solutes beyond ectoine thereby enabling osmolyte switch. This finding thus emphasizes the diverse adaptive strategies employed by halophilic and halotolerant bacteria to thrive in saline environments, aligning with previous reports of glycine betaine production in related halotolerant species such as *B. halodurans* SMBPL06^26^ and *B. subtilis* MA04^48^ thereby implying its importance as one of the reportedly most predominant solutes produced besides ectoine in true halophiles studied till date^49^.

The ability of ectoine-compatible solute production by halophilic bacteria belonging to *Halomonas* species is evidenced by comprehensive reports on ectoine production in many other *Halomonas* species, such as *H. elongate*^50–52^, *H. boliviensis*^53^ and halotolerant bacteria such as *Bacillus halodurans*^25^. Likewise, the production of glycine betaine has also been reported in halotolerant bacteria such as *B. halodurans*^26^, *B. subtilis*^48^, etc. The accumulation of these compatible solutes has been reported to confer osmotolerance in plants. The hyperosmotic tolerance conferred by the genetic transformation of the ectoine biosynthetic gene has already been reported in many plants, such as tobacco^54,55^ and tomato plants^56^. Similarly, the salt tolerance conferred by transforming the glycine betaine biosynthetic gene has been reported in barley^57^ and wheat^58^. The ability of the isolates to produce these compatible solutes thus showed their significance as a source of osmoprotectant responsive genes, which hold a tremendous potentiality for conferring osmotolerance to plants through their genetic transformations.

The application of compatible solutes in various industries for stabilizing enzymes suggests a possible correlation that the ectoine-compatible solute produced by the isolates could be involved in aiding the production of extremozymes under saline conditions by preventing their denaturation from salinity and thereby maintaining their production. Enzymes like protease, cellulase, chitinase, etc. are produced and secreted by halophiles to acquire nutrients and energy from organic substrates present in their extreme environments. However, the high salt concentrations in their environments can disrupt the structure and function of these enzymes by interfering with their electrostatic interactions and hydrogen bonding. So, to counteract these denaturing effects of salt, halophiles have evolved to produce these compatible solutes not only as part of their adaptation to saline environments but also to protect and stabilize their metabolically and physiologically important enzymes for survivability. These compatible solutes are said to form protective hydration shells around the enzymes, shielding them from the disruptive effects of salt ions. This hydration stabilizes the enzyme's structure and allows it to remain active and functional in the presence of high salt. Notably, the observations made by Roberts^20^ and Detkova et al.^21^ affirm this correlation between compatible solutes and extremozymes production. They documented the multifaceted role of compatible solutes in halophilic bacteria, emphasizing not only their pivotal function in osmoregulation to maintain cellular osmotic equilibrium but also their significant capacity to serve as effective stabilizers of proteins and even whole cells. These findings collectively underscore the intimate connection between compatible solute accumulation and the production and stability of extremozymes, including proteases, cellulases, and chitinases, in halophilic microorganisms.

## Materials and methods

The investigation was carried out at the “Department of Biotechnology, College of Agriculture, Junagadh Agricultural University, Junagadh” during 2019–2022.

### Isolation of bacteria

The halophilic and halotolerant bacteria were isolated from 15 different soil samples, each approximately 100 g in weight, collected from various crop rhizospheres in different agricultural fields lying along the southwest coastline of Saurashtra, Gujarat. Specifically, samples were obtained from Junagadh and Porbandar districts, located at coordinates 21.52° N 70.47° E and 21°37′48″ N 69°36′0″ E, respectively, as outlined in Table 4, as previously reported by Reang et al*.*^11^. Following the streak plate method, the bacteria were isolated from 10 ml of soil suspensions (prepared from 1 g) by streaking a loopful of the 10^–5^ dilution onto a freshly prepared autoclaved halophilic agar media supplemented with 10% NaCl, adjusted to pH 7.2 ± 0.2 (at 25 °C), and incubated at 37 °C for 5 days. The isolates were characterized for halophilic and halotolerant nature by subjecting them to a varying concentration of NaCl ranging from 5, 10, 15, 20, and 25% in halophilic broth for salt tolerance test. Pure culture plates of the above isolates were prepared on the same media and used to prepare primary inoculum seed culture.

**Table 4:** Details of soil sampling sites and locations.

Sr. no.	Sampling site	Crop rhizosphere	District	Sr. no.	Sampling site	Crop rhizosphere	District
1	Mangrol	Chilli	Junagadh	9	Madhavpur Ghed	Soyabean	Porbandar
2	Kankana	Cluster bean	Junagadh	10	Gorsar	Pigeon pea	Porbandar
3	Chankhva	Sorghum	Junagadh	11	Untada	Okra	Porbandar
4	Mekhdi	Maize	Junagadh	12	Navi Bandar	Cotton	Porbandar
5	Kalej	Indian bean	Junagadh	13	Tukada	Groundnut	Porbandar
6	Azak	Cowpea	Junagadh	14	Odadar	Brinjal	Porbandar
7	Divasa	Spine gourd	Junagadh	15	Porbandar	Black gram	Porbandar
8	Shil	Smooth gourd	Junagadh	–	–	–	–

### Preliminary soil analysis

The preliminary analysis of soil samples was conducted to assess soil chemical properties, including soil pH using potentiometry and electrical conductivity via the conductometry method^59^. Soil organic carbon content was determined using the back titration method^60^, while available soil phosphorus was measured using a colorimetric method^61^, and soil potash was analyzed via flame photometry^59^.

### Preparation of inoculum

A primary inoculum of the isolates was prepared by inoculating a single colony from each pure culture plate as prepared above on a freshly prepared autoclaved 10 ml halophilic broth in test tubes and incubated at 37 °C for 24 h. As described below, the primary inoculum was then used as seed culture for the extracellular enzymes and compatible solutes production potentiality experiments.

### Microscopic characterization of isolates

#### Gram’s staining

A thick smear of all cultures was prepared on a clean glass slide individually and subjected to the gram staining process. Subsequently, the slides were allowed to air dry at room temperature overnight and observed under the Zeiss Imager.Z2 optical microscope to ascertain the orientation of the isolates.

#### Scanning electron microscopy

A loopful of the bacterial isolates colony was picked from their respective fresh culture plates, and a light smear was made on the aluminium stub with the help of inoculating needle. The smeared stub was then flooded with 4% glutaraldehyde and kept in a fridge at 4 ℃ for 24 h. The following day the smeared samples were dehydrated by using a gradient dilution of acetone in a concentration ranging from 30, 50, 70, 80, 90, and 100% and treating each sample by dipping into dilution of each respective concentration in the order of 30–100% for 15 min. The samples treated by dipping in an acetone concentration of 100% were repeated for a second time for another 15 min for each sample. The dehydrated samples were then coated in a spotter coater with a gold–palladium mixture plate and observed under a scanning electron microscope (Zeiss EVO 18).

#### Motility test

The bacterial motility test was done by the hanging drop method. A few drops of liquid culture were placed onto the coverslip sterilely. A depression slide was taken, and the concave portion over the drop pressed the slide onto the cover slip. The slide was inverted quickly to keep from disrupting the drop. Then the motility was examined under the Zeiss Imager.Z2 optical microscope at 40× magnification.

#### Molecular identification of bacterial isolates

The identification of halophilic and halotolerant bacterial isolates was performed using BLAST analysis of their partial 16S rRNA gene sequences against known reference strains in the curated 16S database of EzBioCloud. Reference strains exhibiting a sequence similarity threshold above 96% with the query isolates were selected for further analysis. A phylogenetic tree was constructed using MEGA11, applying the maximum composite likelihood substitution model with 100 bootstrap replications. The tree was generated under the minimum evolution method to elucidate the genetic interconnectedness among various genera within their respective species.

### Protease enzyme production

The protease enzyme production was carried out by inoculating 1% of the test isolates in a 250 ml Erlenmeyer flask containing 100 ml autoclaved protease production broth prepared by dissolving 3% nutrient gelatin, 0.8% nutrient broth, 0.5% casein, 0.01% MnCl_2_, 15% NaCl, and 1.2 ml of 20% glycerol and incubated at 37 ℃ for 72 h in a shaker incubator (150 rpm). After 72 h of growth, the cells were harvested at 10,000 rpm for 15 min, and the supernatant thus obtained was used as crude enzyme for quantitative assay.

### Protease enzyme quantification assay

The protease enzyme assay followed Sigma's non-specific protease assay described by Cupp-Enyard^62^. The assay was performed in triplicates in 15 ml test tubes using enzyme extracts from each isolate, with one tube serving as a blank control. In each set of four tubes, 5 ml of 0.65% casein solution was added and equilibrated at 37 °C for 5 min. Then, 1 ml of enzyme extract was introduced to three of the tubes (excluding the blank), mixed, and incubated at 37 °C for 10 min. Tyrosine release and protease activity were measured and compared among test isolates during this incubation period. After 10 min, 5 ml of 110 mM TCA reagent was added to stop the reaction and incubated for 30 min at 37 °C. Concurrently, a series of standard tyrosine dilutions were prepared in six test tubes, with incremental volumes of 1.1 mM tyrosine standard stock solution, precisely measuring 0.00, 0.05, 0.10, 0.20, 0.40, and 0.50 ml, respectively. Subsequently, each standard dilution was adjusted to a final volume of 2 ml by the addition of an appropriate volume of purified water.

After a 30-min incubation, each test solution and blank was filtered using a 0.45 μm polyethersulfone syringe filter to eliminate insoluble components. The resulting 2 ml filtrate from both the test isolates and blanks was transferred to new test tubes. In all the test tubes containing standards and standard blank, 5 ml of sodium carbonate was added, followed immediately by 1 ml of Folin’s reagent to stabilize pH. After observing a cloudy appearance due to the reaction with free tyrosine, the tubes were gently mixed and incubated at 37 °C for 30 min. After incubation, the tubes with standards were seen with a gradation of color correlating with the amount of tyrosine added. This color change was also observed in tubes with test samples. Subsequently, 2 ml of these solutions were filtered into cuvettes using a 0.45 μm polyethersulfone syringe filter. The absorbance of the standards, standard blank, the different test isolates, and test blank were measured in a spectrophotometer at 660 nm wavelength with a 1 cm light path. The standard tyrosine curve was then constructed to determine the amount of tyrosine released and estimate the protease activity of the test isolates using the below-described formula.

One unit of protease activity (U) was defined as the amount of enzyme capable of releasing 1.0 μ mol of tyrosine per min from the casein substrate under the described reaction conditions. The protein content was estimated by Lowry’s method, with bovine serum albumin (BSA) as the standard. The protease enzyme and specific activity were then determined by calculating using the following formula:

$${\text{Protease activity }}\left( {{\text{U ml}}^{{ - {1}}} {\text{min}}^{{ - {1}}} } \right) \, = \frac{{\mu {\text{ mol of tyrosine equivalents released }} \times { 11}}}{{\left( {{1 } \times { 1}0 \, \times { 2}} \right)}}$$

where, 11 = Total volume of assay (ml). 10 = Time of assay (min) as per the unit definition. 1 = Volume of enzyme used (ml). 2 = Volume taken in cuvette for colorimetric determination.

$${\text{Protease specific activity }}\left( {{\text{U mg}}^{{ - {1}}} {\text{min}}^{{ - {1}}} } \right) \, = {\text{ Enzyme activity }}\left( {{\text{U ml}}^{{ - {1}}} {\text{min}}^{{ - {1}}} } \right) \, /{\text{ Protein content }}\left( {{\text{mg ml}}^{{ - {1}}} } \right)$$

### Cellulase enzyme production

The cellulase enzyme production was carried out by inoculating 1% of the bacterial isolates in a 250 ml Erlenmeyer flask containing 100 ml autoclaved cellulase production broth prepared by dissolving 1% CMC, 0.2% NaNO_3_, 0.05% MgSO_4_, 0.005% K_2_HPO_4_, 0.001% FeSO_4_, 0.002% CaCl_2_ and MnSO_4_, 15% NaCl, and incubated on water bath shaker at 120 rpm at 37 °C for 5 days for cellulase enzyme production. After incubation, the bacterial cultures were harvested by centrifugation at 5000 rpm for 20 min. The culture supernatants were used for the quantification of the cellulase enzyme.

### Cellulase enzyme quantification assay

The cellulase activity was assayed using the DNSA method, followed by Lay Mg Mg et al*.*^63^. One milliliter of culture supernatant was mixed with 1 ml of 0.05 M citrate buffer (pH 4.8) solution in test tubes containing 1% cellulose substrate. The resulting reaction mixture was incubated at 50 °C for 60 min in a water bath shaker at 80–85 rpm. After the reaction time, 3 ml of DNSA reagent was added to the reaction mixture and boiled for exactly 5 min to terminate the reaction in a vigorously boiling water bath. The reaction mixture was then cooled in a cold water bath, and the absorbance was measured by a spectrophotometer at 540 nm against the blank without enzyme filtrate. Anhydrous glucose was the standard^64^. One unit of cellulase activity (U) was defined as the amount of enzyme capable of releasing 1.0 mg of glucose per min from the cellulose substrate under the described reaction conditions. The protein content was estimated as described above. The cellulase enzyme activity and specific activity were then determined by calculating using the following formula:

$${\text{Cellulase activity }}\left( {{\text{U ml}}^{{ - {1}}} {\text{min}}^{{ - {1}}} } \right) \, = \frac{{\left( {{\text{mg of glucose released }} \times { 5 } \times \, 0.{5}} \right)}}{{\left( {{1 } \times { 2 } \times { 6}0} \right)}}$$

where, 5 = Total volume of assay (ml). 0.5 = Dilution factor (DF). DF = (Vol^m^ of enzyme extract/Vol^m^ of enzyme + buffer). 1 = Volume of enzyme extract used (ml). 2 = Volume of reaction mixture taken in cuvette (ml). 60 = Incubation time (min)

$${\text{Cellulase specific activity }}\left( {{\text{U mg}}^{{ - {1}}} {\text{min}}^{{ - {1}}} } \right) \, = {\text{ Enzyme activity }}\left( {{\text{U ml}}^{{ - {1}}} {\text{min}}^{{ - {1}}} } \right) \, /{\text{ Protein content }}\left( {{\text{mg ml}}^{{ - {1}}} } \right)$$

### Chitinase enzyme production

The chitinase enzyme production was performed by modifying the standard method followed by Hsu and Lockwood^65^. One milliliter of the bacterial isolates was inoculated in the 250 ml Erlenmeyer flask containing 100 ml autoclaved Minimal Medium (Designated as MM) broth prepared by dissolving 0.5% colloidal chitin, 0.05% MgSO_4_∙7H_2_O, 0.03% KH_2_PO_4_, 0.07% K_2_HPO_4_, 0.0001% MnCl_2_, 0.001% FeSO_4_∙7H_2_O and 0.0001% ZnSO_4_, 15% NaCl in 1000 ml distilled water and the final pH was adjusted to 7. The inoculated tubes were incubated in a shaker incubator (200 rpm) at 30 °C for 48 h. After incubation, the isolates' cultures were harvested and used for carrying out further quantitative chitinase enzyme assay. An uninoculated test tube containing the same liquid broth was kept as blank.

### Chitinase enzyme quantification assay

Chitinase activity was determined by modifying a colorimetric method followed by Setia and Sohorjono^66^ in triplicates. The reaction mixture consisted of 1 ml of the crude enzyme and 2 ml of 1.25% (w/v) colloidal chitin substrate in a 200 mM potassium phosphate buffer (pH 6.0). The mixture was incubated at 30 °C for 2 h and boiled for 10 min to stop the reaction, then cooled to room temperature in a cold water bath, and 1 unit of β-*N*-Acetylglucosaminidase (NAGase) was added and then centrifuged at 8000 rpm for 20 min. The 1 ml of test supernatant obtained from the above centrifugation was added to 1.5 ml of freshly prepared color reagent solution prepared by mixing 96 mM DNSA (3,5-Dinitrosalicylic Acid) reagent in 5.3 M sodium potassium tartrate solution and diluted to 40 ml with deionized water. The test supernatant and color reagent solution mixture was then boiled for 5 min and cooled to room temperature. The concentration of GlcNAc (*N*-acetylglucosamine) released was then measured at 540 nm. The standard curve of GlcNAc was plotted between GlcNAc concentration and GlcNAc absorbance. One unit of chitinase enzyme activity (U) was defined as the amount of enzyme capable of liberating 1.0 mg GlcNAc per hour from the chitin substrate under reaction conditions.

The protein content in isolates was determined by the Folin-Lowry method using BSA as standard. Data on chitinase enzyme activity, specific activity, and protein content was analyzed with a single factorial CRD analysis of variance (*α* = 0.05).

$$\begin{gathered} {\text{Calculations}}: \hfill \\ {\text{Standard}}\;{\text{ curve}}: \hfill \\ \Delta {\text{A54}}0{\text{nm}}\;{\text{ Standard }} = {\text{ A54}}0{\text{nm Std }} - {\text{ A54}}0{\text{nm Std Blank}} \hfill \\ {\text{The }}\Delta {\text{A54}}0{\text{nm of the standards was plotted against the milligrams of NAG released}}. \hfill \\ {\text{Sample determination}}: \hfill \\ \Delta {\text{A54}}0{\text{nm Sample }} = {\text{ A54}}0{\text{nm Test }} - {\text{ A54}}0{\text{nm Test Blank}} \hfill \\ \end{gathered}$$

The milligrams of NAG liberated were determined using the standard curve, and the chitinase enzyme activity (U ml^−1^ h^−1^) and its specific activity (U mg^−1^ h^−1^) defined per mg of protein estimated in isolates were then calculated using the following formula:

$${\text{Chitinase activity }}\left( {{\text{U ml}}^{{ - {1}}} {\text{h}}^{{ - {1}}} } \right) \, = \frac{{\left( {\text{mg NAG released}} \right) \, \left( {{3 } + {\text{ Volume of NAGase}}^{{3}} } \right)}}{{\left( {{2 } \times { 1 } \times { 1}} \right)}}$$

where, 3 = Initial reaction volume of assay. 2 = Conversion factor for converting 2 h to 1 h as per the unit definition. 1 = Volume (ml) of supernatant used in colorimetric determination. 1 = Volume (ml) of crude enzyme used. Volume of NAGase = 0.5 ml.

$${\text{Chitinase specific activity }}\left( {{\text{U mg}}^{{ - {1}}} {\text{h}}^{{ - {1}}} } \right) \, = {\text{ Enzyme activity }}\left( {{\text{U ml}}^{{ - {1}}} {\text{h}}^{{ - {1}}} } \right) \, /{\text{ Protein content }}\left( {{\text{mg ml}}^{{ - {1}}} } \right)$$

### Ectoine production potentiality

#### Culture medium

The ectoine production of the bacterial isolates was carried out in an autoclaved freshly prepared culture medium consisting of yeast extract (86 gl^−1^), ammonium sulfate (28 gl^−1^), FeCl_2_.4H_2_O (0.5 mM), MnSO_4_∙H_2_O (10 μM), KCl (2 gl^−1^), MgSO_4_.7H_2_O (100 mM), and sodium chloride (1 M) as per the modified method of Chen et al*.*^47^.

#### Growth conditions

A primary inoculum seed culture of all the isolates was first prepared by inoculating a loopful of the respective halophilic bacterial cells to 10 ml of autoclaved freshly prepared halophilic broth and incubated in a shaker incubator at 37 ℃ and 180 rpm for 24 h. After 24 h cultivation, 1 ml of the seed culture was inoculated into a 250 ml Erlenmeyer flask containing 50 ml autoclaved freshly prepared ectoine production broth and incubated at 30 ℃ and 200 rpm for 24 h. The flask uninoculated with isolate culture served as the negative control. After cultivation, the samples were taken to analyze cell growth and ectoine concentration.

#### Ectoine quantification assay

The halophilic bacterial isolates culture cultivated above was harvested by centrifugation at 8000 rpm. The pellets were resuspended in 80% ethanol (v/v) (Sigma) with vigorous shaking for 30 min. The ethanol extracts were filtrated through a 0.45 mm filter to analyze ectoine production. The ectoine concentration was then determined from the filtrate obtained by LCMS as per the details in Table 5. Ectoine, purchased from Sigma, was used as the reference standard.

**Table 5:** Details of LCMS conditions used for quantification of ectoine.

Sr. no.	LC parameter		Sr. no.	QTOF parameter	
1	Mobile phase		1	Ion-polarity	Positive MS mode
(i)	A	Water with formic acid			
(ii)	B	Methanol with formic acid	2	Source	Dual AJS ESI
2	Flow	1 ml min^−1^	3	Gas Temp	350 °C
3	Time (min)	B	4	Drying GAS	10 l min^−1^
(i)	0	10	5	Nebulizer	40 psi
(ii)	10	50	6	Sheath Gas Temperature	300 °C
(iii)	11	10	7	Sheat Gas Flow	12 l min^−1^
(iv)	13	10	8	V cap	4000 V
4	Injection vol^m^	20 μl	9	Fragmentor	150 V
5	Total run time	13 min	10	Skimmer	65 V
6	Column Temperature	40 °C			

### PCR-based molecular detection of ectoine and glycine betaine biosynthetic genes

The isolates' production potentiality of the ectoine-compatible solute was confirmed by PCR-based molecular detection of its biosynthetic gene *ectC* which encodes for the ectoine synthase enzyme. Besides, the PCR-based molecular screening also showed the presence of *BADH1* gene encoding for betaine aldehyde dehydrogenase enzyme which is responsible for the biosynthesis of glycine betaine compatible solute. The primer sequences for PCR amplification of these genes were obtained from literature reported by Rajan et al.^25^ and Anburajan et al*.* ^26^ for ectoine and glycine betaine, respectively. The isolates' genomic DNA was isolated by Qiagen's blood and tissue kit based on the manufacturer's instructions. The isolated genomic DNA was then analyzed on 0.8% agarose gel electrophoresis, quantified by nanodrop spectrophotometer, and used for further PCR reactions. The PCR reaction was carried out in an Applied Biosystems thermal cycler in a 20 ml reaction volume system containing 50 ng of genomic DNA, 0.5 mM of each primer, 100 mM of each dNTP, 5 U of KAPA Taq DNA polymerase, and 10X Taq A buffer supplemented with 25 mM MgCl_2_. A single PCR vial containing all the above PCR reaction mixture except the DNA was used as the no template control (NTC) to check the chances of amplification due to primer contamination with the template DNA. The thermal cycler amplification reaction conditions were set with an initial denaturation at 94 ℃ for 5 min, followed by 35 cycles of denaturation at 94 ℃ for 45 s, annealing temperature at 50 ℃ for 45 s, and primary extension at 72 ℃ for 1 min, followed by a final extension at 72 ℃ for 7 min and hold at 4 ℃. The PCR amplified products were checked by running on agarose gel electrophoresis at 90 V cm^−1^ in 1.5% low EEO agarose gel prepared in 1× TAE buffer and added with 3% of 1000 ppm ethidium bromide. The resulting electrophoresed amplicons of the respective genes were then scanned and captured by the gel documentation system.

### Statistical analysis

The above experiments were carried out in triplicate replications. The data obtained from their mean values were used for statistical analysis of variance (ANOVA) using a Completely Randomized Design (CRD) for the interpretation of results.

## Conclusion

In the present study, it was thus concluded that the halophilic and halotolerant bacteria isolated from the soils of agricultural fields lying along the southwest coastline of Saurashtra, Gujarat, showed a promising potentiality for production of the industrially important proteolytic, cellulolytic, and chitinolytic extremozymes and may have potential application in many industries especially cellulose and chitinous biomass conversion for biofuel production, etc. Besides, the isolates also represent a potent source of biocontrol agents. They may contribute to sustainable agriculture as an alternative to chemical pesticides against the control and management of fungal diseases, insect pests, and nematodes. However, the isolated halophilic and halotolerant bacteria's reported activities only hint at the possibility of such novel applications. Further study on the candidate isolates’ improvement for higher extremozyme production, their extraction, purification, characterization, and application trials are required for a detailed evaluation of their practical applications. Furthermore, the isolates also exhibited an appreciable potential for producing ectoine-compatible solute, as validated by the presence of its biosynthetic gene. Besides, the isolates also showed the presence of glycine betaine-compatible solute biosynthetic gene. Thus, these isolates may serve as a promising source of osmoprotectant-responsive genes for developing osmotolerant transgenic plants against salinity, heat, and drought stresses, as already reported by many scientists. The study also suggests that there may be a correlation between compatible solutes and extremozyme production of the isolates under saline conditions. The compatible solutes could be playing a vital role in aiding the maintenance of normal extremozyme production by protecting them from salt-induced denaturation effects, potentially enhancing their stability and activity. However, this hypothesis is purely our assumption, and further investigation is required to confirm it.

### Supplementary Information

Supplementary Figure 1.

Supplementary Figure 2.

Supplementary Figure 3.

Supplementary Figure 4.

Supplementary Figure 5.

Supplementary Figure 6.

Supplementary Tables.

## Data Availability

The main author hereby declares with the consent of all concerned co-authors that data and materials related to the work described would only be made available at request to the corresponding author.
