# Supplementary figures and images for: Extremozymes and compatible solute production potential of halophilic and halotolerant bacteria isolated from crop rhizospheric soils of Southwest Saurashtra Gujarat
Source: Sci Rep. 2024 Jul 8;14:15704. doi: 10.1038/s41598-024-63581-z (PMC11231302; doi:10.1038/s41598-024-63581-z)

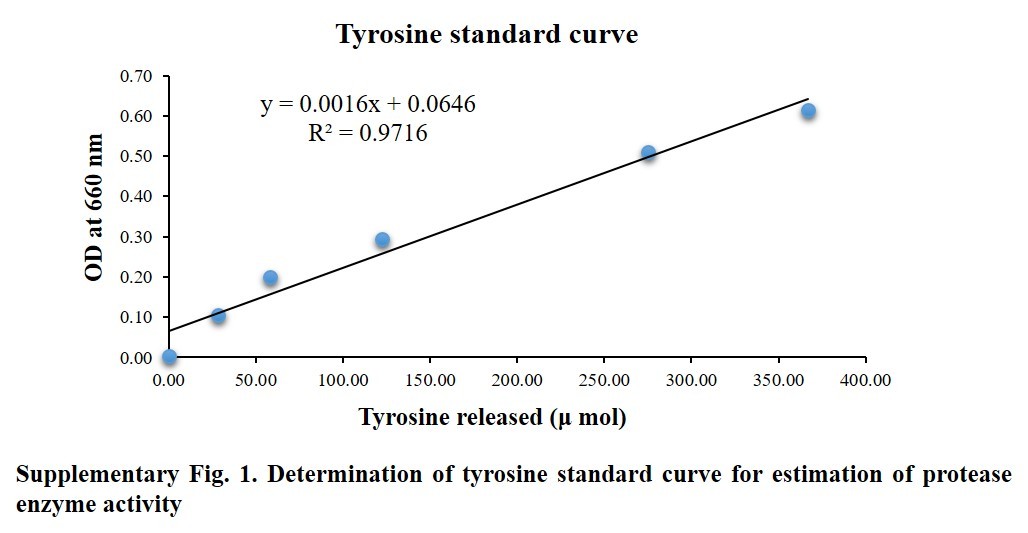

Supplement: Supplementary file 1 — Supplementary Figure 1. [file 41598_2024_63581_MOESM1_ESM.jpg]

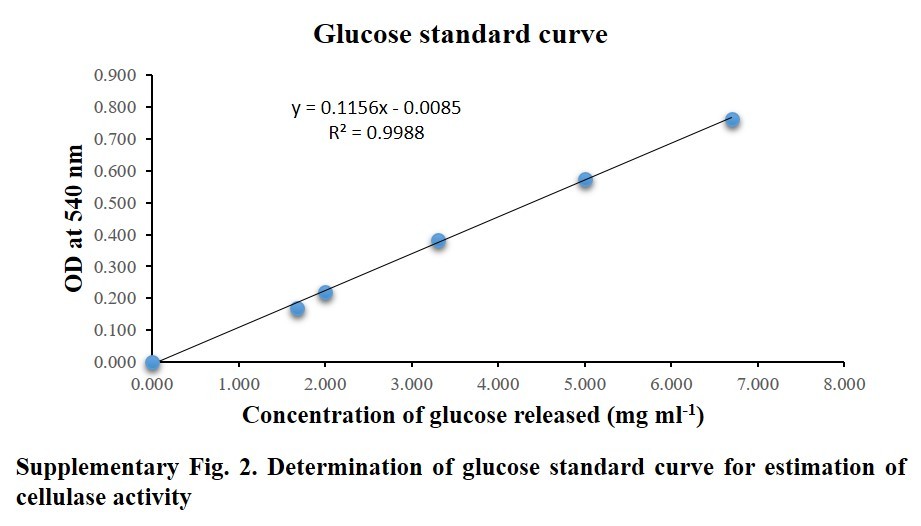

Supplement: Supplementary file 2 — Supplementary Figure 2. [file 41598_2024_63581_MOESM2_ESM.jpg]

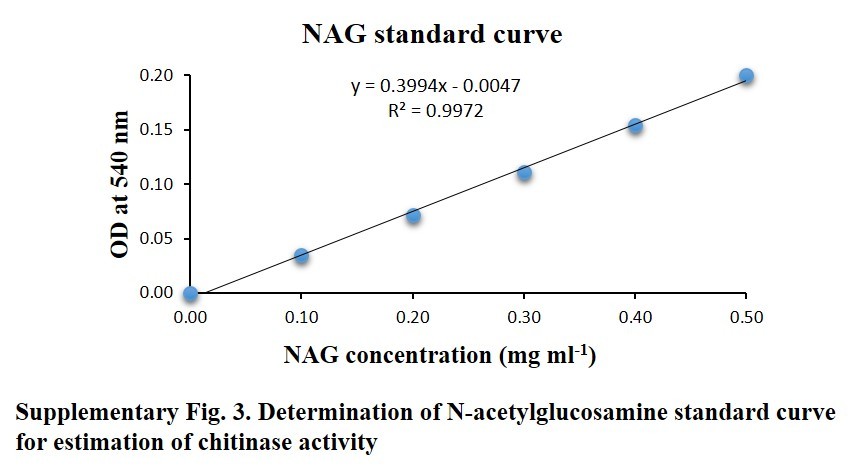

Supplement: Supplementary file 3 — Supplementary Figure 3. [file 41598_2024_63581_MOESM3_ESM.jpg]

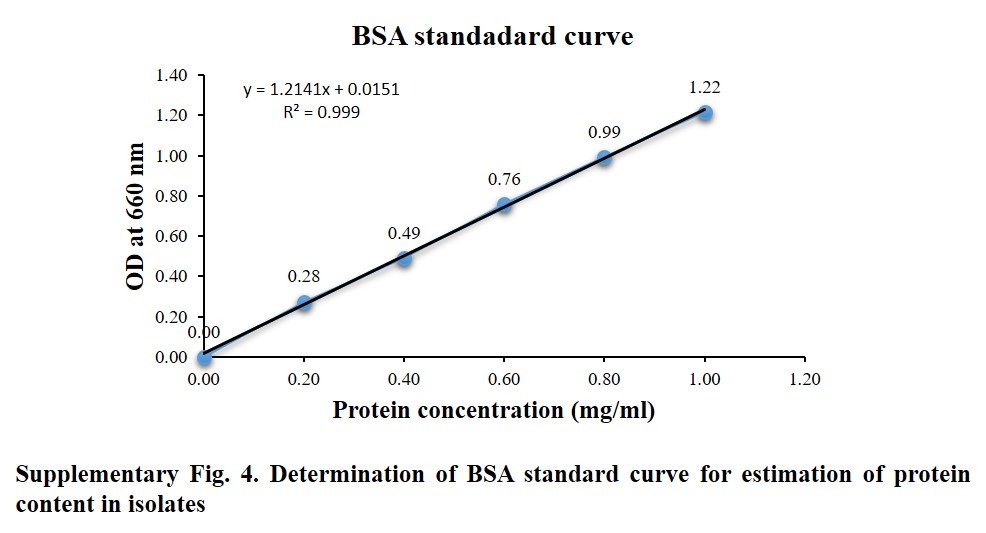

Supplement: Supplementary file 4 — Supplementary Figure 4. [file 41598_2024_63581_MOESM4_ESM.jpg]

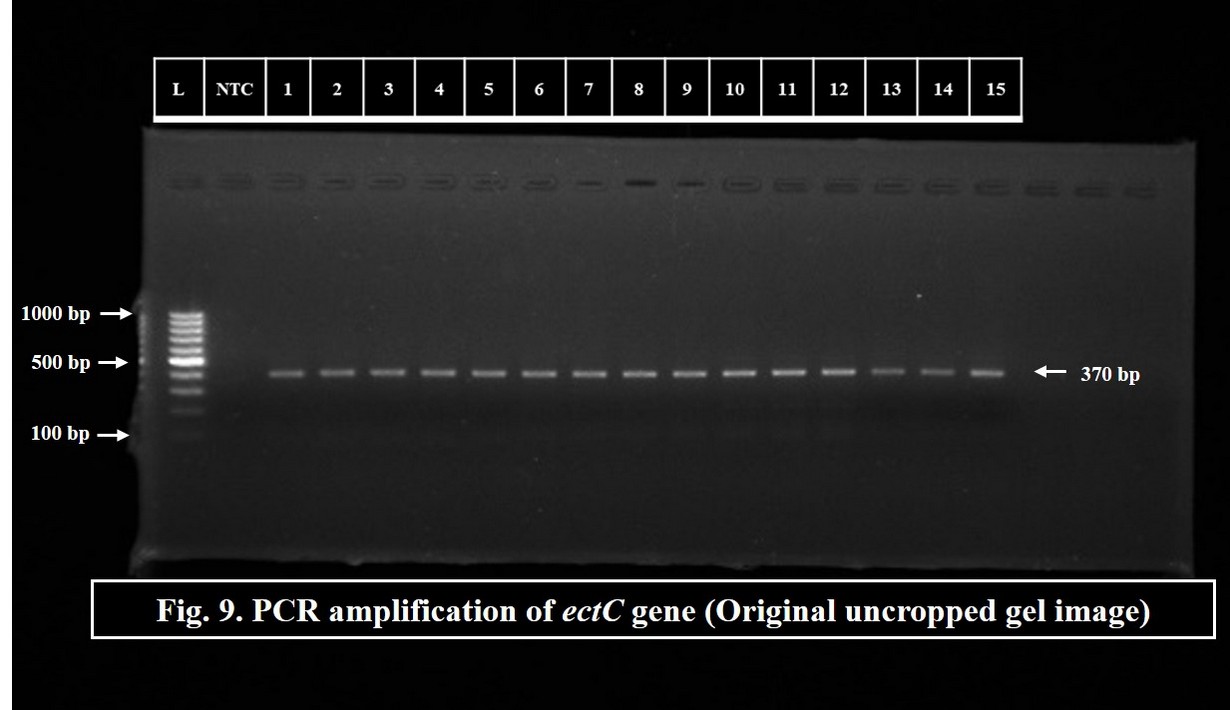

Supplement: Supplementary file 5 — Supplementary Figure 5. [file 41598_2024_63581_MOESM5_ESM.jpg]

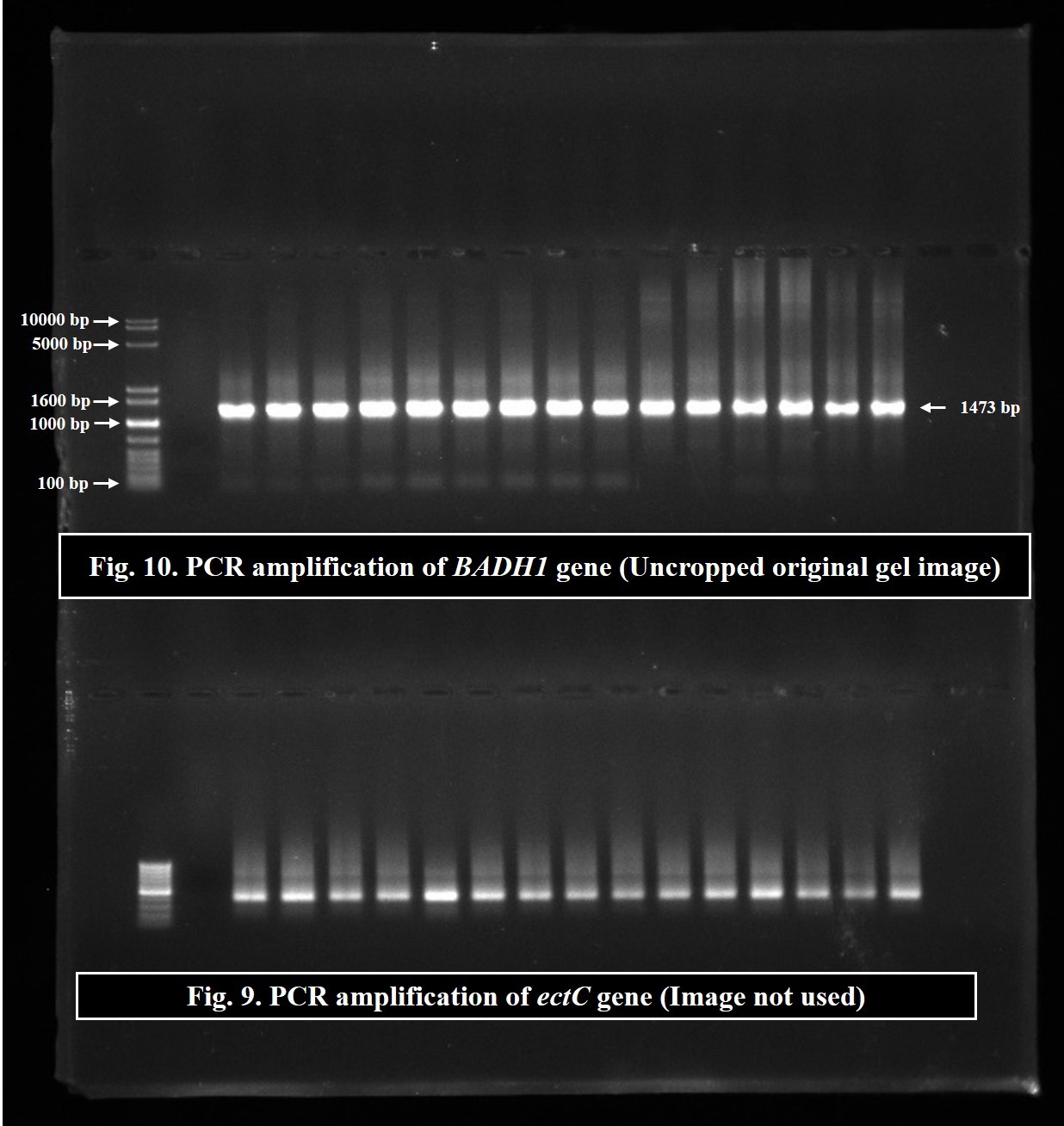

Supplement: Supplementary file 6 — Supplementary Figure 6. [file 41598_2024_63581_MOESM6_ESM.jpg]
