# Supplementary material for: Extremozymes and compatible solute production potential of halophilic and halotolerant bacteria isolated from crop rhizospheric soils of Southwest Saurashtra Gujarat
Source: Sci Rep. 2024 Jul 8;14:15704. doi: 10.1038/s41598-024-63581-z (PMC11231302; doi:10.1038/s41598-024-63581-z)
Supplement: Supplementary file 7 — Supplementary Tables. [file 41598_2024_63581_MOESM7_ESM.pdf]

Supplementary Table 1. Determination of protease enzyme and specific activity of isolates

Sr. No.	Isolate code	Tyrosine released (μ mol)	Protease activity (U ml^{-1} min^{-1})	Protein content (mg ml^{-1})	Specific activity (U mg^{-1} min^{-1})
1.	S1	28.023 ± 0.560	15.413 ± 0.308	0.939 ± 0.003	16.416 ± 0.378
2.	S2	38.521 ± 0.307	21.186 ± 0.169	0.872 ± 0.002	24.296 ± 0.185
3.	S3	16.646 ± 0.364	9.155 ± 0.200	0.797 ± 0.001	11.488 ± 0.267
4.	S4	12.556 ± 0.156	6.906 ± 0.086	0.955 ± 0.0004	7.231 ± 0.087
5.	S5	33.667 ± 0.488	18.517 ± 0.268	0.918 ± 0.001	20.171 ± 0.290
6.	S6	22.412 ± 0.371	12.327 ± 0.204	0.980 ± 0.001	12.578 ± 0.198
7.	S7	25.974 ± 0.340	14.286 ± 0.187	0.876 ± 0.0004	16.308 ± 0.207
8.	S8	15.264 ± 0.390	8.395 ± 0.215	0.854 ± 0.005	9.834 ± 0.312
9.	S9	36.260 ± 0.057	19.943 ± 0.032	0.992 ± 0.008	20.106 ± 0.130
10.	S10	42.088 ± 0.279	23.148 ± 0.154	0.905 ± 0.018	25.595 ± 0.427
11.	S11	20.992 ± 0.442	11.545 ± 0.243	0.890 ± 0.008	12.979 ± 0.383
12.	S12	56.461 ± 0.153	31.054 ± 0.084	0.915 ± 0.005	33.940 ± 0.166
13.	S13	64.317 ± 0.474	35.375 ± 0.261	1.284 ± 0.028	27.586 ± 0.814
14.	S14	53.549 ±0.550	29.452 ± 0.303	0.970 ± 0.017	30.388 ± 0.750
15.	S15	58.836 ± 0.594	32.360 ± 0.327	0.896 ± 0.032	36.205 ± 1.292
16.	Control	0.000	0.000	0.000	0.000
S.Em.±		0.357	0.196	0.010	0.364
C.D. at 5 %		1.029	0.565	0.029	1.048
C.V. %		2.122	2.118	2.111	3.750

Keys: S.Em: Standard Error of mean, C.D.: Critical Difference and C.V.: Coefficient of variation.

Supplementary Table 2. Determination of cellulase enzyme and specific activity of isolates

Sr. No.	Isolate code	Glucose released (mg ml^{-1})	Cellulase activity (U ml^{-1} min^{-1})	Protein content (mg ml^{-1})	Specific activity (U mg^{-1})
1.	S1	0.446 ± 0.011	0.009 ± 0.0003	0.526 ± 0.005	0.018 ± 0.0004
2.	S2	0.528 ± 0.010	0.011±1.23 x 10^{-19}	0.450 ± 0.007	0.024 ± 0.001
3.	S3	0.242 ± 0.010	0.005 ± 0.000	0.494 ± 0.005	0.010 ± 0.001
4.	S4	0.404 ± 0.016	0.008 ± 0.0003	0.572 ± 0.006	0.015 ± 0.001
5.	S5	0.198 ± 0.015	0.004 ± 0.0003	0.596 ± 0.006	0.007 ± 0.001
6.	S6	0.343 ± 0.017	0.007 ± 0.001	0.602 ± 0.007	0.012 ± 0.001
7.	S7	0.244 ± 0.016	0.005 ± 0.001	0.545 ± 0.007	0.009 ± 0.001
88.	S8	0.406 ± 0.010	0.008 ± 0.0003	0.490 ± 0.008	0.017 ± 0.001
9.	S9	0.491 ± 0.009	0.010 ± 0.0003	0.610 ± 0.006	0.017 ± 0.0004
10.	S10	1.167 ± 0.002	0.024 ± 2.45 x 10^{-19}	0.597 ± 0.007	0.041 ± 0.0004
11.	S11	0.432 ± 0.013	0.009 ± 0.000	0.574 ± 0.005	0.016 ± 0.001
12.	S12	1.642 ± 0.011	0.034 ± 0.0003	0.552 ± 0.008	0.062 ± 0.001
13.	S13	2.021 ± 0.013	0.042 ± 0.0003	0.908 ± 0.004	0.046 ± 0.001
14.	S14	1.283 ± 0.018	0.027 ± 0.0003	0.515 ± 0.008	0.052 ± 0.001
15.	S15	1.510 ± 0.016	0.032 ± 0.0003	0.601 ± 0.005	0.052 ± 0.001
16.	Control	0.000	0.000	0.000	0.000
S.Em.±		0.012	0.0003	0.006	0.001
C.D. at 5 %		0.035	0.001	0.017	0.002
C.V. %		3.402	4.215	2.095	4.876

Keys: S.Em: Standard Error of mean, C.D.: Critical Difference and C.V.: Coefficient of variation.

Supplementary Table 3. Determination of chitinase enzyme and specific activity of isolates

Sr. No.	Isolate code	NAG released (mg ml^{-1})	Chitinase activity (U ml^{-1})	Protein content (mg ml^{-1})	Specific activity (U mg^{-1})
1	S1	0.057 ± 0.001	0.154 ± 0.004	0.761 ± 0.011	0.202 ± 0.003
2	S2	0.084 ± 0.001	0.223 ± 0.002	0.604 ± 0.003	0.369 ± 0.001
3	S3	0.037 ± 0.001	0.105 ± 0.002	0.550 ± 0.016	0.190 ± 0.003
4	S4	0.044 ± 0.001	0.124 ± 0.003	0.720 ± 0.004	0.172 ± 0.004
5	S5	0.072 ± 0.001	0.191 ± 0.003	0.752 ± 0.011	0.254 ± 0.004
6	S6	0.052 ± 0.001	0.142 ± 0.003	0.795 ± 0.038	0.180 ± 0.008
7	S7	0.034 ± 0.001	0.097 ± 0.002	0.665 ± 0.008	0.146 ± 0.004
8	S8	0.063 ± 0.001	0.170 ± 0.003	0.698 ± 0.014	0.244 ± 0.009
9	S9	0.104 ± 0.001	0.273 ± 0.003	0.780 ± 0.028	0.351 ± 0.016
10	S11	0.057 ± 0.001	0.156 ± 0.003	0.648 ± 0.012	0.240 ± 0.0003
11	S12	0.177 ± 0.001	0.456 ± 0.004	0.991 ± 0.011	0.460 ± 0.009
12	S13	0.215 ± 0.002	0.550 ± 0.005	1.166 ± 0.003	0.471 ± 0.004
13	S14	0.122 ± 0.002	0.318 ± 0.004	1.054 ± 0.016	0.301 ± 0.005
14	S15	0.157 ± 0.001	0.404 ± 0.004	1.020 ± 0.009	0.396 ± 0.003
15	Control	0.000	0.000	0.000	0.000
S.Em.±		0.001	0.003	0.016	0.006
C.D. at 5 %		0.004	0.009	0.045	0.018
C.V. %		2.486	2.437	3.615	4.080

Keys: S.Em: Standard Error of mean, C.D.: Critical Difference and C.V.: Coefficient of variation.
